# Benchmarking microbiome transformations favors experimental quantitative approaches to address compositionality and sampling depth biases

**DOI:** 10.1038/s41467-021-23821-6

**Published:** 2021-06-11

**Authors:** Verónica Lloréns-Rico, Sara Vieira-Silva, Pedro J. Gonçalves, Gwen Falony, Jeroen Raes

**Affiliations:** 1 Laboratory of Molecular Bacteriology, Department of Microbiology and Immunology, Rega Institute, KU Leuven, Leuven, Belgium; 2 Center for Microbiology, VIB, Leuven, Belgium; 3 Max Planck Research Group Neural Systems Analysis, Center of Advanced European Studies and Research (caesar), Bonn, Germany

**Keywords:** Data processing, Standards, Microbiome

## Abstract

While metagenomic sequencing has become the tool of preference to study host-associated microbial communities, downstream analyses and clinical interpretation of microbiome data remains challenging due to the sparsity and compositionality of sequence matrices. Here, we evaluate both computational and experimental approaches proposed to mitigate the impact of these outstanding issues. Generating fecal metagenomes drawn from simulated microbial communities, we benchmark the performance of thirteen commonly used analytical approaches in terms of diversity estimation, identification of taxon-taxon associations, and assessment of taxon-metadata correlations under the challenge of varying microbial ecosystem loads. We find quantitative approaches including experimental procedures to incorporate microbial load variation in downstream analyses to perform significantly better than computational strategies designed to mitigate data compositionality and sparsity, not only improving the identification of true positive associations, but also reducing false positive detection. When analyzing simulated scenarios of low microbial load dysbiosis as observed in inflammatory pathologies, quantitative methods correcting for sampling depth show higher precision compared to uncorrected scaling. Overall, our findings advocate for a wider adoption of experimental quantitative approaches in microbiome research, yet also suggest preferred transformations for specific cases where determination of microbial load of samples is not feasible.

## Abstract (web summary)

Here, the authors use simulated quantitative gut microbial communities to benchmark the performance of 13 common data transformations in determining diversity as well as microbe-microbe and microbe-metadata associations, finding that quantitative approaches incorporating microbial load variation outperform computational strategies in downstream analyses, urging for a widespread adoption of quantitative approaches, or recommending specific computational transformations whenever determination of microbial load of samples is not feasible.

## Introduction

Metagenomic sequencing allows profiling of microbial communities at an unprecedented scale and throughput. Circumventing cultivation biases, sequencing approaches hold the promise of enabling true random sampling of complex microbial ecosystems. Continuous standardization efforts are being made to minimize systematic biases such as those created by the impact of extraction and amplification procedures on metagenomic readouts^1^. However, even when minimizing technical biases, the analysis and interpretation of current state-of-the-art metagenomes is not exempt of challenges^2–4^.

A first challenge of current practices in metagenomics arises from the proportional nature of the sequencing data generated. Protocols for sequencing library preparation have been optimized to assure maximal sequencing success rates^5^. Extraction procedures are geared towards maximum yields, even when starting from low cell density material, while equimolar pooling of DNA extracted from a broad range of samples assures equal sequencing library sizes across experimental setups. However, while facilitating automation and standardizing data quality, these procedures do not conserve any link between the cell density of the sampled community and the amount of sequencing data generated. Hence, the resulting sequence matrices can only be analyzed in terms of relative proportions of microbial features (taxa or functions) present in a sample of an unquantified community^3^. Within such proportional data structures, relative abundances are not independent (data compositionality). Relative changes in abundance of a single taxon or metabolic pathway are inherently compensated by an equivalent increase/decrease of the remaining feature space, inducing a negative correlation bias^3,6,7^. This limitation affects all downstream microbiome analyses. Proportional analyses cannot characterize the magnitude and directionality of between-sample differences in microbiome composition or metabolic potential. Moreover, when applied to communities displaying substantial variation in ecosystem density (microbial load; Table 1), data compositionality hampers the identification of correlation patterns between microbiome features and with environmental or clinical covariates^8^.

**Table 1:** Glossary of terms.

Microbial load	Also referred to as cell counts or cell density. The number of microbial cells per gram of sample.
Sequencing depth	In this context, sequencing depth or library size is the number of reads obtained for each sample by sequencing (and after filtering-out low quality reads).
Sampling depth	Represents the fraction of cells present in a sample that was actually surveyed and corresponds to the ratio of sequencing depth over total microbial load, assuming equal probability of sequencing any microbial cell.
Discordant associations	Associations detected in both a synthetic community and derived (transformed) sequence matrix, but with inversed directionality. Discordant associations are counted both as false positives in precision and false positive rate calculations and false negatives in sensitivity calculations.
Sensitivity	Proportion of associations present in a synthetic community that are recovered in the derived (transformed) sequence matrix. Calculated as (true positives)/(true positives + false negatives).
Precision	Proportion of associations recovered in a (transformed) sequence matrix that correspond with associations present in the original synthetic community. Calculated as: (true positives)/(true positives + false positives).
False positive rate	Proportion of non-associated features present in a synthetic community that are detected as associated in the derived (transformed) sequence matrix. Calculated as (false positives)/(false positives + true negatives).

Definitions of the technical terms used throughout this study.

A second, related challenge is linked to the low and variable sampling depth that is inherent to current metagenomics sequencing approaches. In microbiome research, sampling depth should be defined as the ratio between the number of cells (partially) sequenced (observed population size) and the total microbial load present in the sample analyzed (true population size) – as opposed to sequencing depth, which corresponds to the amount of sequencing data generated for a single sample^4^ (Table 1). While technical innovations and decreasing sequencing costs have resulted in a gradual increase of sequencing depths over the years, metagenomic analyses remain characterized by shallow sampling depths (Table 1). A recent shotgun metagenomic analysis of 876 fecal samples revealed that sequencing depths ranging between 5.5 and 18.2 Gbp resulted in a 0.0045% average sampling depth (here defined based on the number of cells present in a gram of fecal material)^9^. When assessing dense, diverse, and unevenly distributed microbial ecosystems such as those encountered in stool samples, shallow sampling depths contribute to the sparsity of the resulting microbiome feature matrices and do not allow to distinguish species or function absence from non-detection. Between-sample variation in sampling depth complicates matters even more. Ideally, even when analyzing proportional composition, the surveyed fraction of a microbial community should be kept constant across samples^4^. When assessing communities with varying microbial loads across different samples, maintaining or creating an even sequencing depth independently of sample density generates sequence matrices at uneven sampling depths. Within the *N* = 876 survey of fecal metagenomes discussed above, actual sampling depths were shown to vary more than 40-fold, between 0.0009% and 0.0407% across individuals. Such variation implies that detection of specific microbiome features in a subset of samples could potentially result from uncontrolled variation in sampling depth. Hence, while it is common knowledge that metagenomic analyses never allow determining feature absence, it is less recognized that they might as well identify a mere technical artifact as presence.

The growing appreciation of the severe impact of compositionality and undersampling on metagenomic analyses has led to the active development of a wide range of potential mitigation strategies, both computational and experimental in nature. Microbiome researchers first set out to deal with the issues related to varying sequencing depths – often erroneously considered to be equivalent to sampling depths (i.e. assuming equal microbial densities across the communities sampled)^10,11^. Confronted with technical variation as well as the overall increase in raw sequencing data generated per sample over the years, rarefaction (or downsampling) was suggested to standardize within and across dataset comparisons. In contrast with straightforward normalization to proportional (relative) abundances, random subsetting of the data to even sequencing depth allowed comparing samples in terms of observed richness across samples, independently of the original amount of sequences generated. However, sequencing depth-based downsizing procedures were soon criticized, not only for being wasteful and discarding information on low-abundant taxa^2^, but also for being unsuited when applied to communities characterized by substantial variation in cell density^4^. In response to this, alternative computational approaches have been put forward, consisting in the implementation of robust data transformation strategies that would not only allow dealing with varying sequencing depths, but also with the limitations of compositional data analyses. These approaches find their origins not only in compositional Aitchison statistics^12^, but also in methods developed for normalization and/or differential feature abundance determination in the context of RNA-sequencing based transcriptome analyses^13,14^, among others^15^. Although such transformations have been suggested to be capable of dealing with uneven sequencing depths without a need for data downsizing, it is unclear to what extent they rely on the assumption that all samples analyzed originate from equally dense communities.

While the application of compositionality-aware computational methods would limit the identification of artefacts (such as negative correlations induced by the relative feature space) as true associations, they do not allow retrieving the information that was lost when cutting the link between the microbial loads of the samples analyzed and the amount of sequencing data generated. To resolve this issue, experimental approaches have been developed recently to keep or recover the link with microbial densities, through DNA or cell spike-ins or parallelization of sequencing with quantitative PCR or flow cytometry enumeration of microbial cells^4,8,16–19^. By transforming proportions into counts, such methods eliminate the limitations of compositional data handling from downstream analyses. On top of experimental differences, quantitative approaches vary in the way they incorporate the microbial loads obtained into downstream analyses. Two approaches can be distinguished. On one hand, absolute count scaling procedures multiply relative sequence matrices with experimentally determined microbial loads^16^, retaining all sequencing information generated. On the other hand, quantitative profiling strategies use a stringent downsizing step, evening sampling depths across datasets by randomly subsampling sequencing data to an optimally low sequence/load ratio – at the cost of discarding sequence information and/or samples with insufficient sampling depth^4,8^. Such sampling depth-based downsizing is thought to avoid over-detection of low-abundant taxa in low density samples, which would affect downstream results of diversity and association analyses. Optionally, the resulting non-compositional (uneven) sequence matrices can be scaled to the absolute counts to obtain quantities expressed in interpretable units (e.g. cells per gram of sample material).

Some recent studies have compared the effects of relative normalizations and transformations dealing with compositionality on the correlation structure of microbiome datasets using both real and simulated data^20^. Quantitative approaches have been proposed to be superior to relative analyses in determining taxon–taxon correlations^4^, but have thus far not been systematically benchmarked against compositional transformations. In addition, as current quantitative transformations require experimental determination of microbial loads, they are not compatible with all types of sample collection methods (e.g. the use of stabilization buffers without weight recording) and have been suggested to be overly labor intensive^21^.

**Fig. 1:**
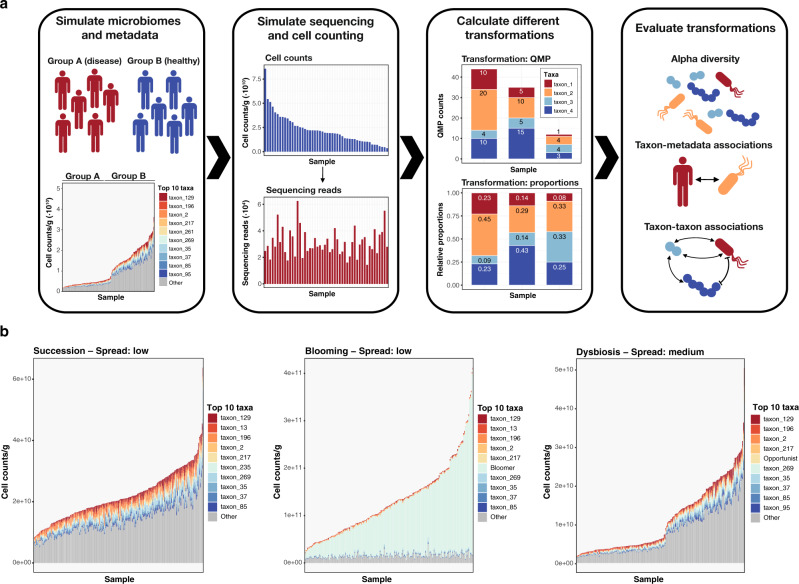
Visualization of study workflow. **a** Study design and approach. First, synthetic microbial communities were designed mimicking three distinct ecological scenarios. Second, metagenomic sequencing of these communities and determination of their microbial loads were simulated. Third, sequencing data were transformed according to a selection of methods being benchmarked. Finally, these methods were evaluated based on their performance in relevant microbiome analyses. **b** Examples of the three types of ecological scenarios simulated: succession (left panel), blooming (mid panel), and dysbiosis (right panel). Samples are ordered in function of microbial density. The top 10 most abundant taxa as well as the bloomer and opportunist taxon are colored for each population.

In this work, to establish whether the game is worth the candle, we perform a systematic evaluation of the advantages and limitations of an extensive set of available computational and experimental transformation approaches that have been proposed to handle compositionality and sampling depth variation in sequence data analyses (Fig. 1a). Our benchmark demonstrates an improved performance of quantitative approaches over other computational methods in reporting sample richness, as well as in accurately recovering true taxon–taxon and taxon–metadata associations, while minimizing the detection of false positive associations.

## Results

Strategies to handle microbiome sequencing data can be broadly classified into four approaches based on their use of untransformed raw sequencing data or the application of relative (simple normalizations), compositional (designed to offset compositionality drawbacks), or quantitative transformations (designed to recover counts); the latter integrating additional experimental data of microbial density (Table 2). To benchmark these data transformation approaches in metagenomic data analysis, we assessed their performance in terms of richness evaluation (alpha diversity), identification of taxon–metadata correlations, and detection of taxon–taxon associations in three distinct ecological scenarios (Fig. 1b and Supplementary Data 1). While framed within human fecal microbiome research, similar scenarios can be expected to occur in a broad range of microbial habitats. A first scenario corresponds to the ecological succession associated with transit time in healthy individuals^22^. Here, richness is observed to increase with fecal microbial loads as transit times are prolonged. In this scenario, most taxon abundances correlate (mildly) positively with microbial cell density. A second scenario represents the blooming of a specific taxon across a population. The bloomer taxon is highly positively correlated with total microbial load, stimulated by selective environmental conditions. Non-bloomer community dynamics remain unaffected, translated in diversification through succession. A third scenario consists of a cohort comprising 50% of patients suffering from a dysbiosis-associated condition as well as 50% of healthy individuals. While succession dynamics drive community variation within the non-patient subgroup, dysbiosis is simulated by reducing overall microbial loads to 20% of eubiotic cell densities through random downsampling^8^, combined with the introduction of both an opportunistic pathogen (negatively correlated with microbial loads/thriving in dysbiosis) as well as several unresponsive taxa (unaffected by dysbiosis/having a non-significant correlation with microbial load).

**Table 2:** Metagenomic data transformations benchmarked.

Method	Abbreviation	Technique	Transformation	Correction	Rarefaction	Suited for richness calculations
Raw sequencing data	Seq	–	None	–	No	Yes
Relative abundance	Rel	Computational	Relative	Sequencing depth	No	No (0:1 range)
Relative microbiome profiling	RMP	Computational	Relative	Sequencing depth	Yes	Yes
Arcsine square root	AST	Computational	Relative	Sequencing depth	No	No (0:1 range)
Centered log ratio	CLR	Computational	Compositional	Sequencing depth and compositionality	No	No (negative values)
Cumulative sum scaling	CSS	Computational	Compositional	Sequencing depth and compositionality	No	Yes (rounding data)
Relative log expression	RLE	Computational	Compositional	Sequencing depth and compositionality	No	Yes (rounding data)
Upper quantile	UQ	Computational	Compositional	Sequencing depth and compositionality	No	Yes (rounding data)
Trimmed mean of m-values	TMM	Computational	Compositional	Sequencing depth and compositionality	No	Yes (rounding data)
Geometric mean of pairwise ratios	GMPR	Computational	Compositional	Sequencing depth and compositionality	No	Yes (rounding data)
Variance-stabilizing transformation	VST	Computational	Compositional	Sequencing depth and compositionality	No	No (negative values)
Quantitative microbiome profiling	QMP	Experimental	Quantitative	Sampling depth and microbial load	Yes	Yes
Absolute count scaling	ACS	Experimental	Quantitative	Microbial load	No	Yes

Methods are categorized based on the technique applied (computational or experimental), the biases targeted (sequencing depth, sampling depth, compositionality, and/or microbial load), the inclusion of a downsizing step, and their projected suitability for richness estimations. Additionally, for study purposes, methods are broadly labeled as relative, compositional, or quantitative methods.

For all three scenarios, taxa abundances for 10 simulations of synthetic microbial community matrices, each comprising 200 samples and 300 taxa, were generated from multivariate negative binomial distributions with taxa-taxa correlations similar to observed in real fecal microbiome datasets. In accordance with observations in fecal samples^4^, microbial loads were set to range between 1.9 × 10^9^ and 1 × 10^13^ cells per gram of stool. Consistent with the biological scenarios considered, cell density spreads (the ratio between the maximum and the minimum microbial load across all samples of each scenario) varied according to the cross-community dynamics imposed (Supplementary Data 1). Experimental quantification of microbial cell counts was mimicked through the addition of noise to the synthetic community densities, with correlation coefficients between the original and estimated counts ranging between 0.85 and 0.95 (Supplementary Data 1). Metagenomic sequencing was simulated by subsampling with replacement an average of 30,000 reads per sample, regardless of the original cell density. This resulted in a relatively lower sampling depth for samples with higher microbial loads (Supplementary Data 1). The resulting sequence matrices were subsequently processed following 13 distinct computational and experimental transformations, classified into four categories based on the nature of the output generated (untransformed raw sequencing, relative transformations, compositional transformations, and quantitative transformations; Table 2). Next, we assessed the relative performance of the methods on the different ecological scenarios presented above.

### Variation in sampling depth affects observed microbiome richness

**Fig. 2:**
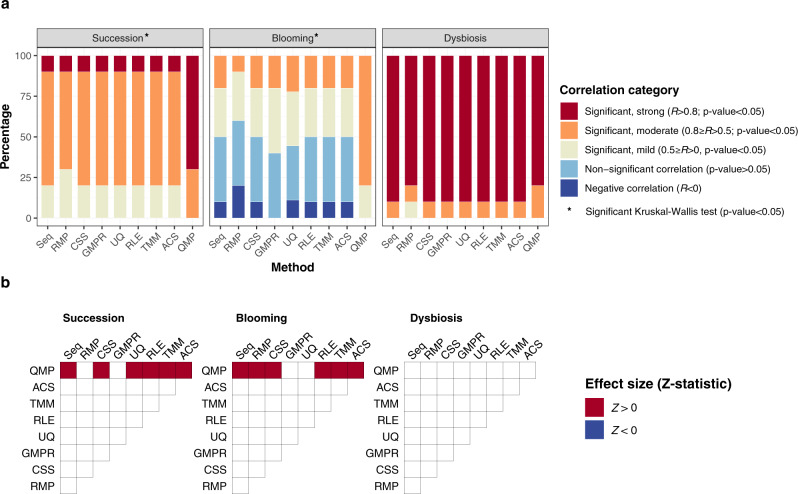
Performance of transformation methods in estimating observed richness. **a** Correlation between the observed richness in the original synthetic communities and (transformed) sequence matrices. Correlations are classified in five categories (two-sided Pearson correlation tests): significant, strong (Pearson *R* > 0.8, *p*-value < 0.05); significant, moderate (0.8 ≥ *R* > 0.5 *p*-value < 0.05); significant, mild (0.5 ≥ *R* > 0, *p*-value < 0.05); non-significant (*p*-value ≥ 0.05); and negative (*R* < 0, *p*-value < 0.05). Stacked barplots represent the percentage of correlations falling into each of the categories for each method and scenario (for *n* = 10 simulated matrices per scenario). Method performance comparison (Pearson *R* median test): $*$, two-sided Kruskal–Wallis *p*-value < 0.05 (Supplementary Data 2). **b** Pairwise evaluation of method performance based on distribution of correlation coefficients determined in panel **a** (Pearson *R* Dunn test, two-sided). Significant (*p*-value < 0.05) comparisons after multiple-testing correction are colored, with colors representing the sign of the effect size (Z-statistic) of each pairwise comparison. A red (blue) color indicates that the method in the corresponding row has a higher (lower) performance than the method in the corresponding column. Seq: sequencing data, RMP: relative microbiome profiling, CSS: cumulative sum scaling, GMPR: geometric mean of pairwise ratios, UQ: upper quartile, RLE: relative log expression, TMM: trimmed mean of M-values, ACS: absolute count scaling, QMP: quantitative microbiome profiling.

First, we evaluated the impact of the transformation methods on the estimated alpha diversities of the communities sequenced. For each scenario, we compared richness and diversity indices of the raw and transformed simulated sequencing data with the readouts of the original synthetic communities (Fig. 2; Supplementary Fig. 1; and Supplementary Data 2). When opting for diversity metrics (Simpson (1-D) or Shannon) reflecting both community richness and evenness, all transformations of the sequencing data matched the original values used for simulation across all scenarios. Minor (∆*R* < 0.01) but significant deviations were observed for methods comprising a downsizing step (i.e. relative microbiome profiling [RMP] and quantitative microbiome profiling [QMP]; Supplementary Fig. 1 and Supplementary Data 2). In contrast, for metrics capturing only microbial richness (observed and estimated [Chao1]), performance varied substantially across methods (Fig. 2; Supplementary Fig. 1; and Supplementary Data 2). In particular, the blooming scenario appeared to be challenging for most approaches: except for QMP, all transformations resulted in a substantial proportion of non-significant or negative associations between richness in the original synthetic communities and in the derived sequence matrices (Fig. 2a). The reason for these aberrant correlations is that in some blooming communities, the increased cell densities (and the inherent decrease in sampling depth) associated with the overgrowth of a single taxon were incorrectly identified by all non-QMP analyses as a reduction of richness (as exemplified in Supplementary Fig. 2a). In fact, whilst in the original blooming communities there is a positive correlation between absolute cell densities and observed richness in spite of the bloomer overgrowth, in both raw and (non-QMP) transformed sequencing data, these correlations become non-significant or negative (Supplementary Fig. 2b). Additionally, in healthy succession and dysbiosis scenarios, all methods except for QMP tended to (moderately) underestimate richness in high cell-count samples (Supplementary Fig. 2a and Supplementary Data 3) – again aligning with the expected decrease in sampling depth. Overall, being the only method correcting for sampling depth variation, QMP performed significantly better in maintaining a moderate to high correlation (*R* > 0.75 in all cases) with actual synthetic community richness when compared to relative and compositional methods, as well as absolute count scaling (ACS; Fig. 2b). Whenever quantitative profiling is technically not possible, diversity indexes (Simpson, Shannon) are robust to compositionality and thus recommended.

### Proportional data blur taxon-load associations

**Fig. 3:**
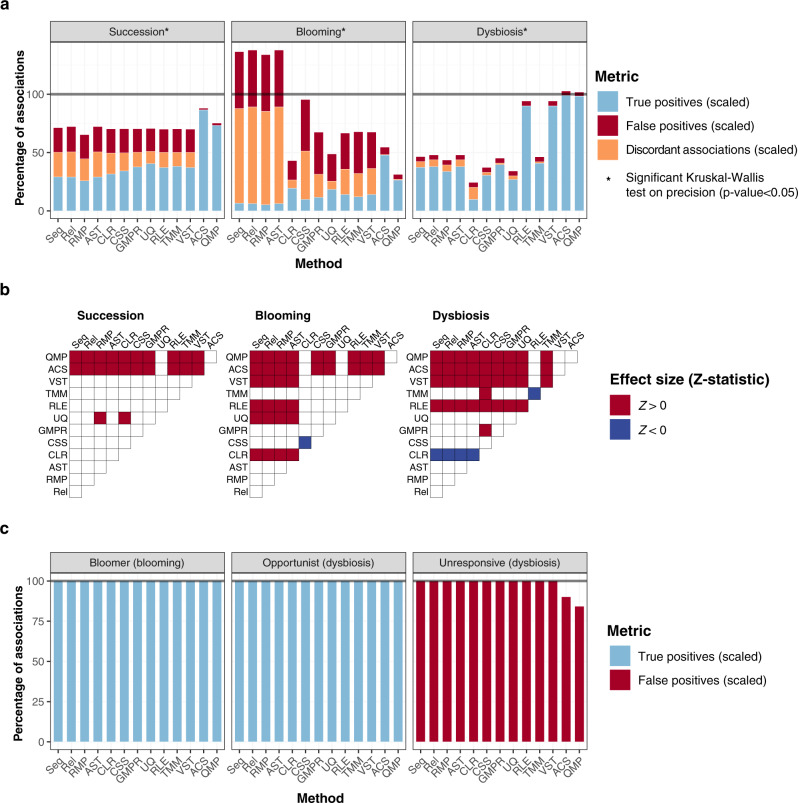
Performance of transformation methods in recovering taxon-load associations. **a** Stacked barplots represent the percentage of true positive, false positive, and discordant taxon-load associations recovered in (transformed) sequence matrices scaled to the number of taxon-load associations present in the synthetic communities for each scenario and method (for *n* = 10 simulated matrices per scenario). Method performance comparison (precision median test): single asterisk, two-sided Kruskal–Wallis *p*-value < 0.05 (Supplementary Data 4). **b** Pairwise evaluation of method precision based on panel **a** classifications (Dunn test, two-sided). Significant (*p*-value < 0.05) comparisons after multiple-testing correction are colored, with colors representing the sign of the effect size (Z-statistic) of each pairwise comparison. A red (blue) color indicates that the method in the corresponding row has a higher (lower) value than the method in the corresponding column. **c** As in **a** for specific taxa in blooming and dysbiosis (bloomer, opportunist, and unresponsive taxa). Bars are scaled to the number of specific taxa in each category (corresponding to the number of potential taxon-load correlations: *n* = 10 for bloomers; *n* = 10 for opportunist taxa and *n* = 104 for unresponsive taxa, considering all the simulated matrices of each scenario altogether). Seq: sequencing data, Rel: relative abundances, RMP: relative microbiome profiling, AST: arcsine square root transformation, CLR: centered log ratio, CSS: cumulative sum scaling, GMPR: geometric mean of pairwise ratios, UQ: upper quartile, RLE: relative log expression, TMM: trimmed mean of M-values, VST: variance-stabilizing transformation, ACS: absolute count scaling, QMP: quantitative microbiome profiling.

Based on previous findings^4^, within the synthetic communities studied, most taxa were coerced to maintain a positive association with total microbial loads. For all scenarios analyzed, this information was largely lost following simulated sequencing and could only be recovered using experimental, quantitative transformations (Fig. 3a). Non-quantitative approaches not only failed to identify a substantial part of taxon-load correlations present in the original synthetic communities, they also led to the detection of a number of false positive and even discordant associations (Table 1), leading to an overall reduction in sensitivity as well as precision (Fig. 3a, b; Supplementary Fig. 3a, b; and Supplementary Data 4). Again, the blooming scenario proved to be most challenging for all methods evaluated. In dysbiosis, the performance of relative log expression [RLE]^13^ and variance-stabilizing transformation [VST] from DESeq2^14^ – normalizations commonly used in the RNA-seq field – was similar to QMP and ACS. While load associations with the bloomer and opportunist taxa were identified correctly by all approaches, the application of non-quantitative methods resulted in an inflation of false positive correlations with the unresponsive taxa present in dysbiosis (Fig. 3c). In a context of decreasing microbial loads, relative and compositional methods misidentify unresponsive taxa as positively associated with dysbiosis, a matter of key importance for biological interpretation in disease-association studies that aim to identify taxa with a potentially causal role in pathogenesis. It should be noted, however, that in this specific case, the burden of false positive detections remained high even in ACS and QMP. Since unresponsive taxa do not display any association with the total microbial loads, these false positives mainly resulted from the noise introduced when simulating experimental quantification of the synthetic community densities (Supplementary Fig. 3c). When analyzing communities with demonstrated stable microbial densities, experimental errors in load quantification could be a significant drawback to be taken in consideration when deciding on a suited strategy for microbiome profiling.

The observed loss of detection of taxon-load associations also impacted the strength of the correlations between taxon absolute abundances as observed in the original synthetic communities and their metagenomic readouts (proportions or counts obtained after sequencing and/or transformation; Supplementary Fig. 4 and Supplementary Data 5). Also here, quantitative methods managed to recover a stronger correlation between the original data and the derived sequence matrices. Again, blooming proved most challenging for all methods evaluated, but particularly for the relative approaches (relative abundances [Rel], relative microbiome profiling [RMP], and arcsine square-root transformation [AST]^23^) that additionally yielded non-significant associations (Supplementary Fig. 4).

### Low cell-count dysbiosis challenges relative and compositional association analyses

The exploration of potential associations between taxa and environmental or clinical parameters is a key aspect of current microbiome research. To assess the impact of computational and experimental transformation procedures on such covariate analyses, we simulated metadata matrices for each of the synthetic microbial communities. Each metadata matrix covered 100 potential microbiome covariates, comprising their corresponding values for the 200 samples in each synthetic microbial community matrix. Out of the 100 metadata variables, 50 were implemented as numeric (following different probability distributions as detailed in Methods) and 50 as categorical. A subset of 20 metadata variables (10 numeric/10 categorical) was created as correlating with one of the 300 taxa, while 10 metadata variables (5 numeric/5 categorical) correlated with sample cell densities.

**Fig. 4:**
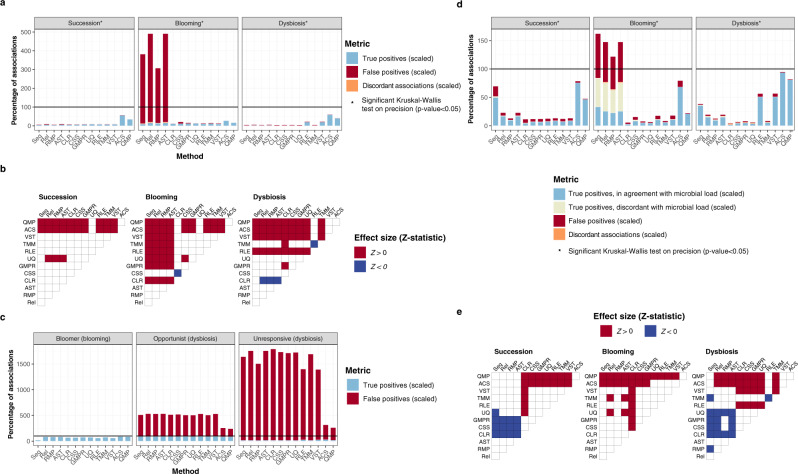
Performance of transformation methods in recovering taxon–metadata and taxon–taxon associations. **a** Stacked barplots represent the percentage of true positive, false positive, and discordant taxon–metadata associations recovered in (transformed) sequence matrices scaled to the number of taxon–metadata associations present in the synthetic communities for each scenario and method (for *n* = 10 simulated matrices per scenario). Method performance comparison (precision median test): single asterisk, two-sided Kruskal–Wallis *p*-value < 0.05 (Supplementary Data 6). **b** Pairwise evaluation of method precision based on panel **a** classifications (Dunn test, two-sided). Significant (*p*-value < 0.05) comparisons after multiple-testing correction are colored, with colors representing the sign of the effect size (Z-statistic) *s* of each pairwise comparison. A red (blue) color indicates that the method in the corresponding row has a higher (lower) value than the method in the corresponding column. **c** As in **a** for specific taxa in blooming and dysbiosis (bloomer, opportunist, and unresponsive taxa; for *n* = 10 simulated matrices per scenario). **d**, **e** As in **a**, **b** for taxon–taxon associations (Supplementary Data 8). Seq: sequencing data, Rel: relative abundances, RMP: relative microbiome profiling, AST: arcsine square root transformation, CLR: centered log ratio, CSS: cumulative sum scaling, GMPR: geometric mean of pairwise ratios, UQ: upper quartile, RLE: relative log expression, TMM: trimmed mean of M-values, VST: variance-stabilizing transformation, ACS: absolute count scaling, QMP: quantitative microbiome profiling.

Taxon–metadata correlations were evaluated for all transformation methods across the three scenarios studied using the associations in the original synthetic communities as a standard (Fig. 4a). Quantitative methods were shown to outperform relative and compositional approaches, displaying significantly higher levels of precision and sensitivity across scenarios (Fig. 4b, Supplementary Fig. 5, and Supplementary Data 6). As was seen for richness and load associations, blooming proved to be the most challenging setting. In this scenario, among non-quantitative methods, compositional transformations yielded significantly higher precision values than relative transformations and raw sequencing (Fig. 4b). These differences could be attributed to the inflation of false positives detected using relative methods (Fig. 4a). Noticeably, when assessing dysbiosis, relative log expression (RLE) and the related variance-stabilizing transformation (VST) performed similar to quantitative methods and superior to other compositional transformations, suggesting that these methods should be chosen in case–control microbiome studies whenever experimental determination of microbial load is not feasible.

Given their potential relevance in clinical studies, we zoomed in on the performance of the different transformation methods with respect to detection of covariation of metadata with the bloomer, the opportunist, and the unresponsive taxa (Fig. 4c and Supplementary Data 7). Since associations between those specific microorganisms and for example disease status could be considered indicative of their potential value as biomarker or therapeutic target, it is of key importance that they are identified accurately. Detection of false positive associations might entice resource- and time-consuming translational follow up of non-relevant targets, with high costs to society. Our results showed that all methods (except the untransformed raw sequencing) identified metadata associated with the bloomer taxon with high sensitivity and precision. In contrast, associations with the (potentially pathogenic) opportunist taxon, thriving in dysbiosis and displaying a negative correlation with microbial load, could only be detected precisely by using quantitative transformations. For the unresponsive taxa in dysbiosis, all methods yielded a large proportion of false positives, although quantitative approaches performed markedly better (Fig. 4c). Overall, these results highlight the need for quantitative methods, especially in case–control studies of disorders associated with microbial dysbiosis, to correctly capture which taxa associate with external metadata relating to disease status, activity, or severity.

### Quantitative methods show highest sensitivity in capturing taxon–taxon associations

In line with metadata-microbiome covariation analyses, we assessed whether the raw sequencing and transformed matrices allowed recovering taxon–taxon associations present in the original synthetic communities. In microbiome research, such associations are often considered indicative of ecological interactions such as cross-feeding, niche sharing, or competition^24^. For the purpose of this evaluation, we mapped all intertaxon associations both in synthetic communities as well as in raw and transformed sequencing data based on Spearman correlation analyses. Again, the quantitative transformations yielded the highest precision values across the ecological scenarios tested (Fig. 4d, e; Supplementary Data 8, and Supplementary Data 9), while centered log-ratio transformation (CLR) appeared least suited for taxon–taxon association analyses. In healthy succession and dysbiosis, ACS and QMP additionally performed best in terms of sensitivity (Supplementary Fig. 6). In contrast, in the blooming scenario, highest sensitivity levels were observed in raw sequence matrices and relative transformations – although at the expense of a high false positive rate (Supplementary Fig. 6). It should be noted, however, that more than half of the true positives generated by these techniques were artefactual, reflecting associations between taxa with discordant load correlations (Fig. 4d). While displaying a positive load correlation in the original synthetic communities, taxa constituting such true positive, discordant associations both exhibited a negative correlation with cell density in raw sequence matrices and following relative transformations. Detection of a positive relationship between such taxa should be considered a true artefact of the methods applied – in this case an artefact that, by pure coincidence, reflected reality.

### Increasing cohort size can affect precision of non-quantitative approaches

Microbiome studies come in a variety of shapes, with an important variation in terms of targeted sequencing depth and cohort size. As these parameters are thought to impact analysis results, we evaluated their effects on performance of the different transformation methods included in the present benchmarking effort. Precision and sensitivity in detection of taxon–metadata and taxon–taxon associations were estimated for all approaches as a function of sequencing depth and cohort size. We observed the sensitivity of quantitative methods regarding both taxon–metadata and taxon–taxon associations to increase with sequencing depth (Supplementary Fig. 7 and Supplementary Data 10), whilst results of relative and compositional approaches were only moderately affected. Variation in cohort size (Supplementary Fig. 8 and Supplementary Data 11) had a both larger and more complex impact on the performance of transformation methods. Regarding taxon–metadata associations, increasing cohort sizes improved sensitivity across all transformations, especially in succession and dysbiosis. In the succession and blooming scenarios, precision remained stable for quantitative approaches, while it decreased with sample size for relative and compositional transformations due to false positive inflation. This means that relative and compositional methods are sensitive to noise introduced by larger cohorts, and results obtained by these methods should be interpreted with care. Similar trends were observed with respect to taxon–taxon associations. The exception here was the increase in precision with cohort size for relative transformations in blooming. However, as discussed above, a large proportion of the true positives in relative transformations is purely artefactual (see Fig. 4d). When using quantitative methods, precision again remained stable independently of cohort size. These results demand careful attention when performing power analyses to determine cohort sizes, as for most methods, an increase of sensitivity is accompanied by an excess of false positives and a decrease in method precision.

### Sampling-depth-based downsizing increases precision when exploring disease-associated low cell-count dysbiosis

The comparison between quantitative methods revealed that the highest sensitivity was reached when keeping the maximum information available (ACS approach) and not rarefying to correct for uneven sampling depths (unlike QMP), both when assessing taxon–taxon and taxon–metadata associations (Fig. 4a, d). These observations question the need for downsizing in quantitative microbiome analyses. The rationale behind the implementation of QMP downsampling procedures is to prevent overrepresentation of rare, possibly unresponsive taxa in low cell density samples, in which sampling depth is higher if similar sequencing depths are achieved across all samples in a population. If these taxa are overly detected in low microbial load samples, it may appear that they are increased and could mistakenly be correlated with metadata features that associate with cell counts (for instance, stool moisture or inflammation in gut microbiome scenarios^8^).

**Fig. 5:**
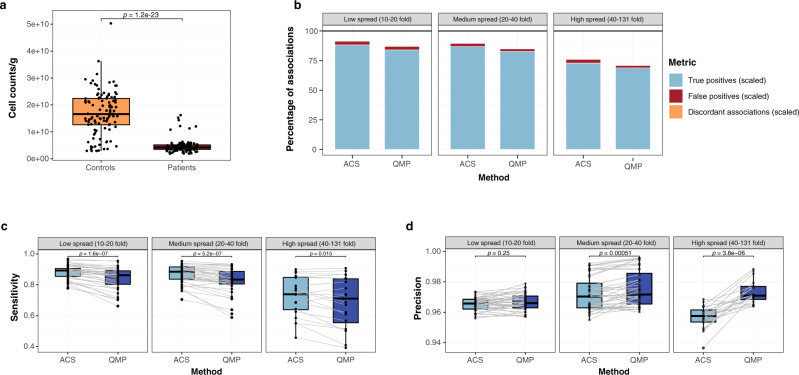
Detection of disease-associated taxa by quantitative methods. **a** Visualization of microbial loads distribution for samples assigned to the simulated patient and control groups in one of the dysbiosis matrices randomly selected (in which *n* = 200 samples, distributed as 93 healthy and 107 diseased; Wilcoxon rank-sum test, two-sided; *p-*adjusted for *n* = 10 matrices). The disease status variable is associated with total cell counts, but noise is present in the dataset. **b** Stacked barplots represent the percentage of true positive, false positive, and discordant taxon-disease associations recovered in ACS- and QMP-transformed sequence matrices at different sequencing depths, scaled to the number of taxon-disease associations present in the synthetic communities. **c** Sensitivity of both methods in detecting taxa-disease associations at different sequencing depths (Wilcoxon signed-rank test, two-sided). **d** Precision of both methods in detecting taxa-disease associations at different sequencing depths (Wilcoxon signed-rank test, two-sided). In **b**–**d**, simulated synthetic community matrices and their derived sequencing data were classified in three groups corresponding to the different spreads in microbial loads in the population (low spread, left panel: *n* = 40 sequencing matrices simulated from 4 synthetic communities; medium spread, mid panel: *n* = 40 sequencing matrices simulated from 4 synthetic communities; high spread, right panel: *n* = 20 sequencing matrices simulated from 2 synthetic communities). In **a**, **c**, **d**, the boxplots extend from the first to the third quartile of the distribution, with the line indicating the median. The whiskers cover from the quartiles to the last data point within 1.5x the interquartile range, with outliers depicted as individual points. ACS: absolute count scaling, QMP: quantitative microbiome profiling.

To further explore these considerations, we focused on the dysbiosis scenario, mimicking a dataset in which a disorder is associated to lower cell densities and creating a disease status metadata variable negatively associated with cell counts (exemplified in Fig. 5a). In this scenario, the majority of taxa correlated to cell counts and therefore positively associated to the healthy group. Additionally, synthetic communities comprised an opportunist taxon associated specifically with disease and 10–11 unresponsive (stable) taxa, uncorrelated with microbial load or health status (Supplementary Fig. 9a). From these dysbiotic synthetic communities, we simulated sequencing data at different depths, ranging from ~10,000 to ~100,000 reads per sample. For each sequence matrix, we computed both quantitative transformations (ACS and QMP) and determined their performance in detecting taxon-disease associations for all taxa (Fig. 5b–d) and focusing only on the opportunist and unresponsive taxa (Supplementary Fig. 9b, c). To evaluate the results, we ranked the original dysbiosis matrices according to their spread in microbial loads. Higher spreads can be expected to lead to larger differences in sampling depths, making the accurate detection of the opportunist and unresponsive taxa more challenging. Our results showed that, in agreement with our previous analyses, ACS displayed higher sensitivity to detect true associations of taxa with the disease covariate, the majority of them comprising species correlating positively with microbial loads and thus more abundant in the healthy group. However, this difference in sensitivity between ACS and QMP decreased with increasing load differences between patients and controls (Fig. 5c), with the effects of sampling depth variation (ACS) and loss of information due to downsizing (QMP) evening out. Both transformation methods allowed to capture correctly the association between the opportunist taxon and the disease (Supplementary Fig. 9b and Supplementary Data 12). Overall precision was higher in QMP, with the performance gap widening with increasing spreads (Fig. 5d). This difference can be attributed to the lower false positive rates observed after sampling depth-based downsizing. When focusing on the unresponsive taxa (without association to the healthy or diseased subpopulation), we found that even at low spreads, QMP resulted in a reduced detection of false positives (Supplementary Fig. 9c). Indeed, lower cell counts, as often observed among patient groups^8^, result in higher sampling depths, leading to a proportional increase in detection of unvarying taxa. Since ACS does not correct for sampling depth, these taxa risk to be erroneously associated with the pathology studied.

Although QMP clearly has an advantage over ACS when analyzing datasets with substantial variation in microbial load, it should be noted that sampling depth downsizing protocols rely on incomplete databases^4^ or, alternatively, on the suboptimal assumption^9^ of equal sequencing probability for any microbial cell. For 16S rDNA amplicon sequencing, sampling depth is estimated based on current knowledge of copy number variation^25^, which is based on a biased collection of fully sequenced reference genomes. For shotgun metagenomics, the number of cells sequenced can be derived more correctly from the abundance of single-copy marker genes^26^. Subsequent downsizing of reads mapping to non-marker gene can be done directly, based on the assumption of similar genome sizes across the species present. Alternatively, genomes sizes can be derived from reference genome databases, provided that they encompass the phylogenetic breadth required for accurate extrapolations across the microbial communities studied. After downsizing and/or quantitative scaling of the sequence matrices, both QMP and ACS facilitate the full range of metagenomic analyses that have become the standard in the field, including quantitative characterization of metagenome-assembled genomes^27^.

## Discussion

Starting from a set of three realistic ecological scenarios, we here benchmarked 13 different analytical approaches for microbiome research to characterize their potential to deal with data compositionality and varying sampling depths, challenges encountered both in amplicon sequencing and shotgun metagenomics. Our results demonstrated that quantitative approaches performed better than their relative and compositional counterparts when it comes to identifying taxon–metadata associations or studying taxon–taxon interactions. The observed performance gap among the methods profiled widened with increasing unevenness of taxa distributions, as exemplified by our analyses of the blooming scenario. Correction for sampling depth, requiring downsizing of metagenomic readouts taking into account microbial loads, proved essential to conserve key community features such as community richness. Although this downsizing resulted in a loss of information and lower sensitivity of quantitative microbiome profiling compared to absolute count scaling, it was accompanied by a lower detection of false positive associations, leading to increased precision. Overall, our findings emphasize the importance of obtaining experimental quantitative data (based on qPCR, DNA or cell spike-ins, cell counts via flow cytometry, or approximated by total microbial DNA quantification) to complement relative sequencing analysis, as current computational alternatives are not sufficiently powered to overcome the compositionality effects. Among quantitative approaches, including a step of downsizing to achieve even sampling depth demonstrated superior performance in the identification of true taxa-disease associations. Whenever microbial load quantification is not possible, computational methods addressing compositionality outperformed relative methods, with only minor differences among them (e.g. superior performance RLE and VST in the dysbiosis scenario). Overall, we recommend that the use of experimental approaches should become standard in current microbiome workflows.

## Methods

### Synthetic microbial communities

Ten synthetic microbial communities were created as matrices of 1000 samples each composed of 300 microbial taxa using Matlab R2015b. Each repeat of 1000 microbiomes was sampled from a model of 300 taxa distributed according to a multivariate negative binomial, generated using Gaussian copulas, with specified correlations among these taxa. In more detail, the procedure followed for each repeat was:

1. Generation of a random matrix of correlations across the 300 taxa, such that the majority of the correlations were positive and a small fraction negative, as is typically observed in empirical data^4^;
2. Generation of 1000 samples from a 300-dimensional multivariate Gaussian distribution centered at zero, with unity variances, and the correlations matrix generated in step 1;
3. Transformation of the matrices obtained in order for the resulting synthetic microbiomes to be distributed according to a multivariate negative binomial, by:
  i Computing the gaussian cumulative distribution for each marginal distribution obtained in step 2;
  ii Computing the negative binomial inverse cumulative distribution for each gaussian cumulative distribution in step 3, with the respective negative binomial parameters (*r*, *q*) generated from uniform distributions *r* ~ U{1,2} and *q* ~ U]0; 0.1[.

### Synthetic microbial communities – ecological scenarios

Three ecological scenarios were derived from each synthetic microbial community: taxon blooming, ecological succession, and taxon opportunism. The above procedure was used to generate the matrices in the taxon blooming scenario. The two other scenarios were derived from the blooming matrices as follows: for each matrix, the bloomer was replaced by a taxon with a specified correlation to the synthetic microbial community loads, while maintaining the rest of the across-taxa correlations matrix. The bloomer taxon was identified as the taxon whose abundance correlated (Spearman *ρ* > 0.9) with synthetic microbial community loads (total community abundance). For the ecological succession scenario, the bloomer was replaced by a taxon with a mild correlation to the synthetic microbial load (succession taxon, specified correlation *r* = 0.5).

For the dysbiosis scenario, half of the synthetic communities, selected by uniform stratification along the microbial loads, were randomly downsampled to 20% of the load, the bloomer was replaced by an opportunistic taxon negatively correlated (specified correlation *r* = −0.5) with microbial loads (thus thriving in dysbiosis), and 10–11 taxa were replaced to be unresponsive towards dysbiosis (specified correlation with synthetic microbial loads *r* = 0.01). Concretely, the succession, opportunist, and unresponsive taxa were created following these steps:

1. Synthetic microbial community loads, assumed to be approximately Gaussian distributed, were *z*-scored to obtained approximately standard Gaussian distributed samples;
2. Standard Gaussian distributed samples were generated with the specified correlations (0.5, −0.5, or 0.01, for the succession, opportunist, and unresponsive taxa, respectively) to the *z*-scored loads obtained in step 1;
3. These Gaussian samples were transformed to be distributed according to a negative binomial, by:
  i Computing the Gaussian cumulative distribution for the samples generated in step 2;
  ii and computing the negative binomial inverse cumulative distribution for the gaussian cumulative distribution in step 3, with the negative binomial parameters *r* = 1 and *q* = 0.5.

For most of the analyses in this work, the matrices were randomly subsampled without replacement to include only 200 samples per matrix. The exception was to analyze the effect of cohort size in the performance of the different transformations, for which other sample sizes, ranging from 20 to 1000 samples per matrix, were used.

### Simulated metadata

For each synthetic microbial community, an associated metadata matrix was generated to obtain absolute Spearman correlation coefficients ranging from 0.3 to 0.6 between the metadata and taxa (or microbial loads). Metadata matrices consisted of 100 metadata features, of which 50 are numeric and 50 are categorical. Numeric variables were (i) generated randomly without specifying correlation with any taxon (*n* = 35/50), from three different distributions: uniform, gaussian or negative binomial, (ii) correlated with any of the absolute taxon abundances in the taxonomic matrix (*n* = 10/50); or (iii) correlated with the absolute microbial loads in the simulated taxonomic matrix (*n* = 5/50). Categorical features were defined as 2, 4, 6, or 8-class random categorical variables. Similarly to numerical variables, these features were defined as: (i) not correlated with any taxon (*n* = 35/50); (ii) correlated with one of the taxa from the taxonomic matrix (*n* = 10/50); or (iii) correlated with microbial loads (*n* = 5/50). Noise was added to avoid perfect associations with categorical features, by first creating a surrogate variable with a mild correlation with the taxon of interest (*ρ* between 0.3 and 0.6) and then the categorical metadata variable was associated to the surrogate variable.

### Metagenomic sequence matrices

Sequence matrices from the original simulated profiles were generated via random sampling with replacement. The number of total reads sequenced per sample was drawn from a log-normal distribution. In the simulations with fixed sequencing depths, the parameters used were log-transformed mean (meanlog = 10.3) and standard deviation (sdlog = 0.3), to reproduce the sequencing depths in a range currently standard in clinical microbiome sequencing experiments (with an average of 30.000 sequenced reads per sample). In the simulations with increasing sequencing depths, the parameter meanlog was taken in the range [9.9, 13.12] to obtain averages of total sequencing depth in the range [20,000, 500,000].

### Estimated microbial loads determination

Estimated microbial loads were calculated by adding noise to the actual microbial loads of synthetic microbial communities, simulating flow cytometry measurements of total cell counts. Flow cytometric quantification of microbial loads has been demonstrated to have a high accuracy when compared to microscopy-based quantification in fresh sedimental samples^28^. Additionally, in stool samples, it has been shown that freeze-thaw cycles have a minor impact in the microbial load quantification, with a correlation of *r* = 0.91 between fresh and frozen fecal aliquots in flow cytometry assays^4^. Therefore, noise was added to the original microbial loads by simulating a correlated vector of estimated microbial loads, such that the Pearson correlation between the original and the estimated microbial loads (in log scale) was in the range [0.85-0.95].

### Normalization and data processing

Different tools and statistical packages were used to preprocess the sequence matrices generated to determine taxon–taxon, taxon–metadata and total counts-metadata correlations. As reference, these correlations were calculated from the original simulated taxonomy matrix.

1. Relative transformations (not addressing metagenomic data compositionality)
  i. Relative microbiota profiling (RMP). RMP matrices were obtained by rarefying all samples in each sequence matrix to an even sequencing depth (the minimum sample total read count of the matrix). This method is used as implemented in the R package phyloseq (v1.34.0)^29^.
  ii. Relative proportions (Rel). Absolute counts from metagenomic sequencing were converted to relative proportions by dividing each taxon abundance by the total taxa abundance in a sample.
  iii. Total sum scaling and arcsine squared transformation (AST). In this method, each taxon count is first scaled dividing by the corresponding sample total counts (total sum scaling, TSS), and the arcsine transform of the scaled values is computed^23^. The method, used in several microbiome research publications^15,30^, was used with a custom implementation. Since it is a direct transformation of the relative proportions (Rel), this method was classified as a relative transformation.
2. Compositional transformations (computational approaches to bypass data compositionality)
  i. Centered log-ratio transformation (CLR). The log ratio of each taxon counts to the geometric mean of all taxa in a sample is computed in this approach. Prior to the transformation, zero’s in the sequence matrix were imputed by Bayesian multiplicative replacement (implemented in the R package zCompositions (v1.3.4)^31^). This method was used as implemented in the CoDaSeq R package (v0.99.6)^6^.
  ii. Cumulative sum scaling (CSS). In this method, taxon counts are divided by the cumulative sum of counts of each sample, up to a percentile determined ad-hoc for each dataset, based on the data distribution. The method was used as implemented in the R package metagenomeSeq (v1.32.0)^32^
  iii. Geometric mean of pairwise ratios (GMPR). This method is used to calculate a scaling factor to normalize the samples. It first computes the median of all pairwise ratios between any two samples, using only non-zero values. The scaling factor of a sample is then calculated as the geometric mean of the median values calculated for that sample and all of the other samples in the dataset. The method was used as implemented in the GMPR R package (v0.1.3)^33^.
  iv. Trimmed mean of M-values (TMM). In this method, the authors defined the M values as the log-ratio between the relative abundance of each gene (or taxon) *g* in a given sample and in a reference sample. To choose a reference sample, the sample whose upper quartile is closest to the mean upper quartile of all the samples tested is used. For each non-reference sample, the M values for all genes/taxa are calculated and the extremes are trimmed. The mean of the remaining *M* values is used as scaling factor for the normalization^34^. The method was used as implemented in the edgeR package (v3.32.1)^13^.
  v. Upper quantile normalization (UQ). For this normalization, scaling factors are calculated from the 75% quantile of the counts for each sample, after removing taxa abundances that are zero, and scaled by sequencing depth. The method was used as implemented in the edgeR package (v3.32.1)^13^.
  vi. Relative log expression (RLE). In this method, the geometric mean of each taxon across all samples is calculated. The median ratio of each sample to the vector of geometric means (excluding zeros) is used as scaling factor for normalization. The method was used as implemented in the edgeR package (v3.32.1)^13^.
  vii. Variance-stabilizing transformation (VST). In this method, taxa counts are scaled by their corresponding library size factors (calculated similarly as in RLE) and a variance-stabilizing transformation is applied that considers the relationship between the dispersion and the mean. The method was used as implemented in the DESeq2 R package (v1.30.1)^14^.
3. Quantitative transformations (experimental approaches to bypass data compositionality)
  i. Quantitative microbiota profiling (QMP). In this method, samples are first rarefied to even sampling depth. Sampling depth, not to be confused with sequencing depth, represents the fraction of the actual observed microbiota in a sample. It can be defined as the ratio between sequencing depth (here taken as the total number of sequencing reads that are assigned to any taxa in a sample) and the total microbial load per gram of the original sample. QMP matrices were generated by rarefying (randomly subsetting) the sequence matrices to even sampling depth considering their synthetic microbial loads, then scaling them by multiplying each sample by its estimated microbial load, as implemented in the original publication^4^.
  ii. Absolute counts scaling (ACS). The ACS matrices were derived as previously reported^7,16^, i.e., by directly multiplying the relative sequencing counts of each sample by their estimated microbial loads.

### Alpha diversity indices

Four different alpha diversity indices were included in this study: observed richness, Chao1 richness estimator, Simpson’s diversity (1-D) and Shannon diversity indices. These were calculated using the implementation provided in the phyloseq R package (v1.34.0)^29^. All the indices were calculated for the simulated microbial communities, the sequenced metagenomes, and all of the transformations calculated, except for the following: the CLR and the VST transformation, which contain negative values that are not accepted by these algorithms; and the Rel and AST transformations, which results only in values between 0 and 1. The remaining transformed matrices were rounded to the closest integer to calculate the different alpha diversity matrices.

### Method evaluation and comparison

Method performance across different microbial loads was evaluated by determining the overall precision (true positives/(true positives + false positives)), sensitivity (true positives/(true positives + false negatives)) and false positive rate (false positives/(false positives + true negatives)) of each transformation tested, taking the associations in the original synthetic microbial communities as a reference. Detected associations are considered significant for *p*-values of the Spearman correlation test <0.05, after correcting for multiple testing with the Benjamini–Hochberg method (BH-corrected *p*-values). Comparison across methods was performed using Kruskal–Wallis tests and post-hoc Dunn tests where applicable, unless otherwise specified. All statistical tests were two-sided. Besides evaluating the overall performance of the transformations in detecting associations, we also detailed their performance on detecting associations corresponding to the special taxa: the bloomer, opportunist and unresponsive taxa. The R tidyverse environment (v1.3.0) and additional packages gdata (v2.18.0), rstatix (v0.7.0), ggpubr (v0.4.0), ggsignif (v0.6.1), inlmisc (v0.5.2), and RColorBrewer (v1.1.2) were used for data wrangling and visualization.

### Reporting summary

Further information on research design is available in the Nature Research Reporting Summary linked to this article.

## Supplementary information

Supplementary Information

Description of Additional Supplementary Files

Supplementary Data 1

Supplementary Data 2

Supplementary Data 3

Supplementary Data 4

Supplementary Data 5

Supplementary Data 6

Supplementary Data 7

Supplementary Data 8

Supplementary Data 9

Supplementary Data 10

Supplementary Data 11

Supplementary Data 12

Reporting Summary

## Data Availability

The simulated data used to evaluate the different data transformations in this manuscript, is available at https://raeslab.org/software/BMT/index.html; and has also been deposited at the Zenodo repository, with the following 10.5281/zenodo.4719508^35^.

## Code availability

The code to generate the taxonomic matrices and their associated metadata, the processed sequencing, RMP, QMP, and ACS matrices, together with the evaluation of the 13 transformation methods, is available at https://raeslab.org/software/BMT/index.html and has also been deposited at the Zenodo repository, with the following 10.5281/zenodo.4719508^35^.

## References

[1] Costea PI (2017). Towards standards for human fecal sample processing in metagenomic studies. Nat. Biotechnol..

[2] McMurdie PJ, Holmes S (2014). Waste not, want not: why rarefying microbiome data is inadmissible. PLoS Comput. Biol..

[3] Gloor GB, Macklaim JM, Pawlowsky-Glahn V, Egozcue JJ (2017). Microbiome datasets are compositional: and this is not optional. Front. Microbiol..

[4] Vandeputte D (2017). Quantitative microbiome profiling links gut community variation to microbial load. Nature.

[5] Yuan S, Cohen DB, Ravel J, Abdo Z, Forney LJ (2012). Evaluation of methods for the extraction and purification of DNA from the human microbiome. PLoS ONE.

[6] Gloor GB, Wu JR, Pawlowsky-Glahn V, Egozcue JJ (2016). It’s all relative: analyzing microbiome data as compositions. Ann. Epidemiol..

[7] Jian C, Luukkonen P, Yki-Järvinen H, Salonen A, Korpela K (2020). Quantitative PCR provides a simple and accessible method for quantitative microbiota profiling. PLoS ONE.

[8] Vieira-Silva, S. et al. Quantitative microbiome profiling disentangles inflammation- and bile duct obstruction-associated microbiota alterations across PSC/IBD diagnoses. *Nat. Microbiol*. **4**, 1826–1831 (2019). 10.1038/s41564-019-0483-9 31209308

[9] Vieira-Silva S (2020). Statin therapy is associated with lower prevalence of gut microbiota dysbiosis. Nature.

[10] Sanders HL (1968). Marine benthic diversity: a comparative study. Am. Nat..

[11] Hughes JB, Hellmann JJ (2005). The application of rarefaction techniques to molecular inventories of microbial diversity. Methods Enzymol..

[12] Aitchison, J. *The Statistical Analysis Of Compositional Data* (Chapman & Hall, 1986).

[13] Robinson MD, McCarthy DJ, Smyth GK (2010). edgeR: a Bioconductor package for differential expression analysis of digital gene expression data. Bioinformatics.

[14] Love MI, Huber W, Anders S (2014). Moderated estimation of fold change and dispersion for RNA-seq data with DESeq2. Genome Biol..

[15] Lloyd-Price J (2019). Multi-omics of the gut microbial ecosystem in inflammatory bowel diseases. Nature.

[16] Props R (2017). Absolute quantification of microbial taxon abundances. ISME J..

[17] Stämmler F (2016). Adjusting microbiome profiles for differences in microbial load by spike-in bacteria. Microbiome.

[18] Tkacz A, Hortala M, Poole PS (2018). Absolute quantitation of microbiota abundance in environmental samples. Microbiome.

[19] Barlow JT, Bogatyrev SR, Ismagilov RF (2020). A quantitative sequencing framework for absolute abundance measurements of mucosal and lumenal microbial communities. Nat. Commun..

[20] Badri, M., Kurtz, Z. D., Müller, C. L. & Bonneau, R. Normalization methods for microbial abundance data strongly affect correlation estimates *BioRxiv*10.1101/406264 (2018).

[21] Morton JT (2019). Establishing microbial composition measurement standards with reference frames. Nat. Commun..

[22] Falony, G., Vieira-Silva, S. & Raes, J. Richness and ecosystem development across faecal snapshots of the gut microbiota. *Nat. Microbiol*. 10.1038/s41564-018-0143-5 (2018). 10.1038/s41564-018-0143-5 29693658

[23] Sokal, R. R. & Rolf, F. J. *Biometry: The Principles and Practice of Statistics in Biological Research* (W. H. Freeman, 1981).

[24] Faust K, Raes J (2012). Microbial interactions: from networks to models. Nat. Rev. Microbiol..

[25] Stoddard SF, Smith BJ, Hein R, Roller BRK, Schmidt TM (2015). rrnDB: improved tools for interpreting rRNA gene abundance in bacteria and archaea and a new foundation for future development. Nucleic Acids Res..

[26] Sunagawa S (2013). Metagenomic species profiling using universal phylogenetic marker genes. Nat. Methods.

[27] Almeida A (2021). A unified catalog of 204,938 reference genomes from the human gut microbiome. Nat. Biotechnol..

[28] Deng L (2019). Improving the accuracy of flow cytometric quantification of microbial populations in sediments: importance of cell staining procedures. Front. Microbiol..

[29] McMurdie PJ, Holmes S (2013). phyloseq: an R package for reproducible interactive analysis and graphics of microbiome census data. PLoS ONE.

[30] Morgan XC (2012). Dysfunction of the intestinal microbiome in inflammatory bowel disease and treatment. Genome Biol..

[31] Palarea-Albaladejo J, Martín-Fernández JA (2015). ZCompositions - R package for multivariate imputation of left-censored data under a compositional approach. Chemom. Intell. Lab. Syst..

[32] Paulson JN, Stine OC, Bravo HC, Pop M (2013). Differential abundance analysis for microbial marker-gene surveys. Nat. Methods.

[33] Chen, L. et al. GMPR: a robust normalization method for zero-inflated count data with application to microbiome sequencing data. *PeerJ* **6**, e4600 (2018). 10.7717/peerj.4600 PMC5885979 29629248

[34] Robinson MD, Oshlack A (2010). A scaling normalization method for differential expression analysis of RNA-seq data. Genome Biol..

[35] Lloréns-Rico, V., Vieira-Silva, S., Gonçalves, P., Falony, G. & Raes, J. *Data Transformations on Si**mulated Microbial Communities*10.5281/zenodo.4719508 (2021).

