# Supplementary material for: Benchmarking microbiome transformations favors experimental quantitative approaches to address compositionality and sampling depth biases
Source: Nat Commun. 2021 Jun 11;12:3562. doi: 10.1038/s41467-021-23821-6 (PMC8196019; doi:10.1038/s41467-021-23821-6)
Supplement: Supplementary file 1 — Supplementary Information [file 41467_2021_23821_MOESM1_ESM.pdf]

Supplementary Information for:

Benchmarking microbiome transformations favors experimental quantitative approaches to address compositionality and sampling depth biases

Verónica Lloréns-Rico[1,2], Sara Vieira-Silva[1,2], Pedro J. Gonçalves[3], Gwen Falony[1,2,*] and Jeroen Raes[1,2,*,§]

[1] Laboratory of Molecular Bacteriology, Department of Microbiology and Immunology, Rega Institute, KU Leuven, Belgium
[2] Center for Microbiology, VIB, B-3000 Leuven, Belgium
[3] Max Planck Research Group Neural Systems Analysis, Center of Advanced European Studies and Research (caesar), Bonn, Germany

[*]Authors contributed equally to this manuscript.
[§]Corresponding author
Mailing address:
Department of Microbiology and Immunology, KU Leuven - Campus Gasthuisberg
Rega Herestraat 49 - box 1028, B-3000 Leuven.
Phone: +32 26 29 13 41 Fax: +32 16 34 60 35
Email: jeroen.raes@kuleuven.be

This file includes:

Supplementary Figures 1-9

Supplementary figure 1

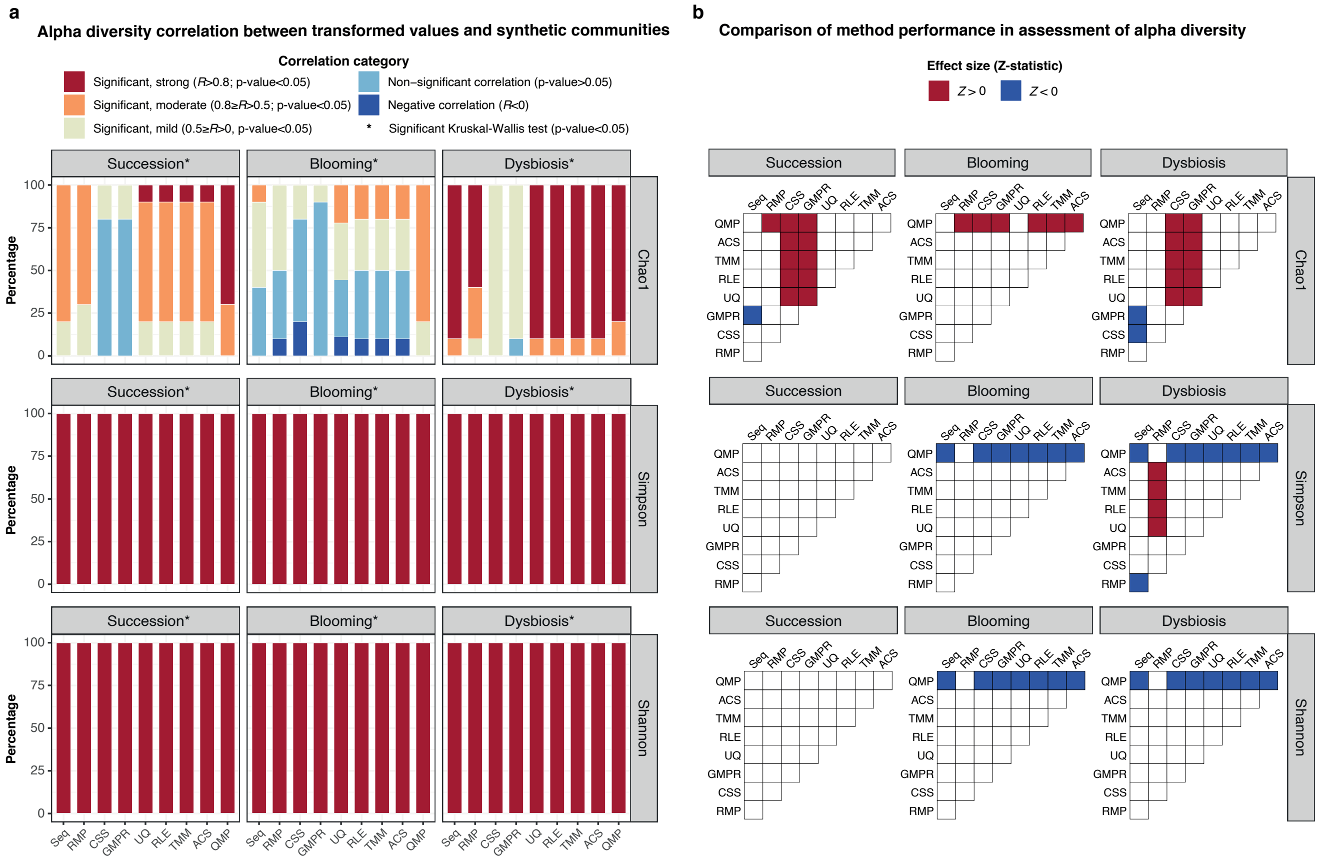

Supplementary Figure 1. Performance of transformation methods in estimating alpha diversity metrics. a. Correlation between the Chao1 (upper panel), Simpson (1-D; mid panel), and Shannon (lower panel) diversity metrics in the synthetic communities and (transformed) sequence matrices (Pearson). Correlations are classified in five categories: significant, strong (*R*>0.8, p-value<0.05); significant, moderate (0.8≥*R*>0.5 p-value<0.05); significant, mild (0.5≥*R*>0, p-value<0.05); non-significant (p-value≥0.05); and negative (0>*R*, p-value<0.05). Stacked barplots represent the percentage of correlations falling into each of the categories for each method and scenario (for *n*=10 simulated matrices per scenario). *, Kruskal-Wallis p-value<0.05 (Supplementary table 2). **b.** Evaluation of method performance based on distribution of correlation coefficients (*R*) determined in panel (a) (pairwise Dunn test). Significant (p-value<0.05) comparisons after multiple testing correction are colored, with colors representing the sign of the effect size (Z-statistic) of each pairwise comparison. A red (blue) color indicates that the method in the corresponding row has a higher (lower) value than the method in the corresponding column. Seq, sequencing data; RMP, relative microbiome profiling; CSS, cumulative sum scaling; GMPR, geometric mean of pairwise ratios; UQ, upper quartile; RLE, relative log expression; TMM, trimmed mean of M-values; ACS, absolute count scaling; QMP, quantitative microbiome profiling.

Supplementary figure 2

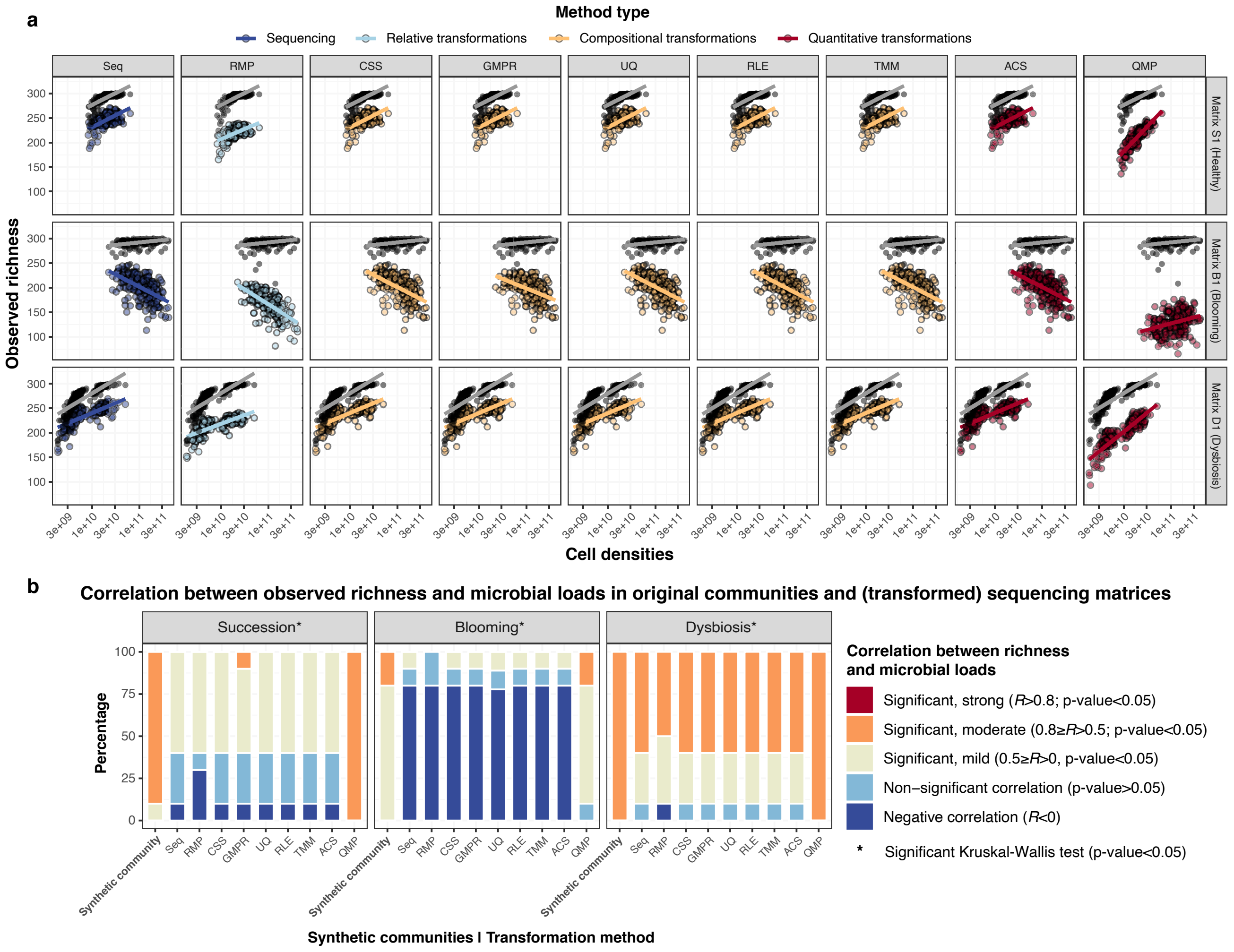

Supplementary Figure 2. Performance of transformation methods in estimating observed richness with varying microbial load. a. Observed richness as a function of microbial loads. Scatter plots show the observed richness as a function of the cell densities (in cells/gram) for a single synthetic community of each scenario. Each scatter plot represents a different method (colored by type of transformation) and a different scenario. A trend line has been added for each of the plots. The grey points and grey trend line in each scatter plot illustrate the observed richness for in the original synthetic communities. **b.** Correlation between observed richness and microbial load in the synthetic communities and (transformed) sequence matrices (Pearson). Correlations are classified in five categories: significant, strong (R>0.8, p-value<0.05); significant, moderate (0.8≥R>0.5 p-value<0.05); significant, mild (0.5≥R>0, p-value<0.05); non-significant (p-value≥0.05); and negative (0>R, p-value<0.05). Stacked barplots represent the percentage of correlations falling into each of the categories for each method and scenario (for n=10 simulated matrices per scenario). *, Kruskal-Wallis p-value<0.05 (Supplementary table 3). Seq, sequencing data; RMP, relative microbiome profiling; CSS, cumulative sum scaling; GMPR, geometric mean of pairwise ratios; UQ, upper quartile; RLE, relative log expression; TMM, trimmed mean of M-values; ACS, absolute count scaling; QMP, quantitative microbiome profiling.

Supplementary figure 3

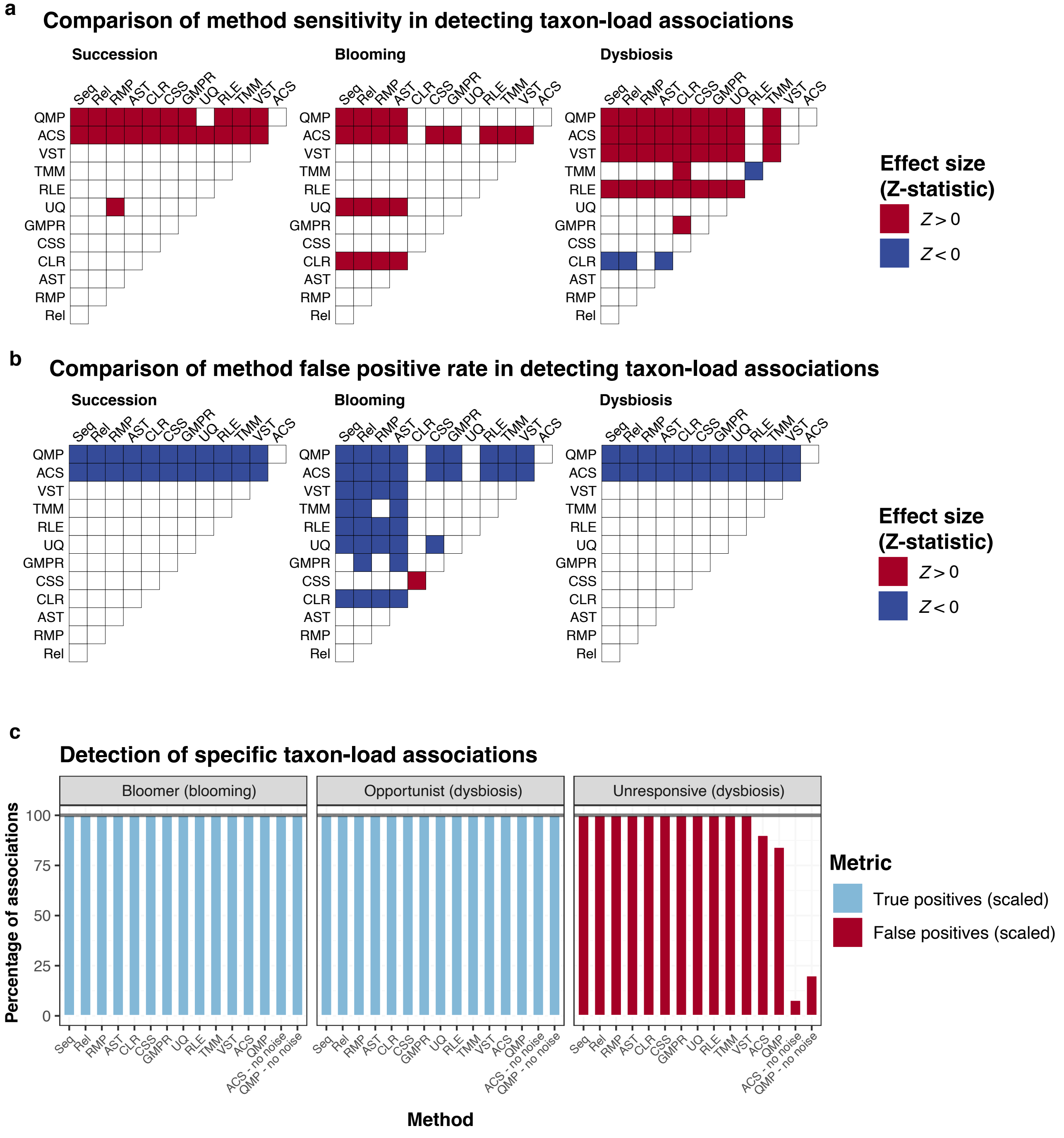

Supplementary Figure 3. Sensitivity and false positive rate of transformation methods in recovering taxon-load associations. a. Evaluation of method sensitivity based on Figure 3a classifications (pairwise Dunn test; Supplementary Table 4). Significant (p-value<0.05) comparisons after multiple testing correction are colored, with colors representing the sign of the effect size (Z-statistic) of each pairwise comparison. A red (blue) color indicates that the method in the corresponding row has a higher (lower) value than the method in the corresponding column. **b.** As in (a) for false positive rates. **c.** Stacked barplots representing the percentage of true positive and false positive

taxon-load associations recovered in (transformed) sequence matrices for specific taxa: the bloomer, the opportunist and the unresponsive taxa (similar to Figure 3c). For quantitative methods (ACS and QMP), the data without noise in the microbial load determination (‘no noise’) has been added for comparison. Bars are scaled to the number of specific taxa in each category (corresponding with the number of potential taxon-load correlations: *n*=10 for bloomers; *n*=10 for opportunist taxa and *n*=104 for unresponsive taxa, considering all the simulated matrices of each scenario altogether). Seq, sequencing data; Rel, relative abundances; RMP, relative microbiome profiling; AST, arcsine square root transformation; CLR, centered log-ratio; CSS, cumulative sum scaling; GMPR, geometric mean of pairwise ratios; UQ, upper quartile; RLE, relative log expression; TMM, trimmed mean of M-values; VST, variance-stabilizing transformation; ACS, absolute count scaling; QMP, quantitative microbiome profiling.

Supplementary figure 4

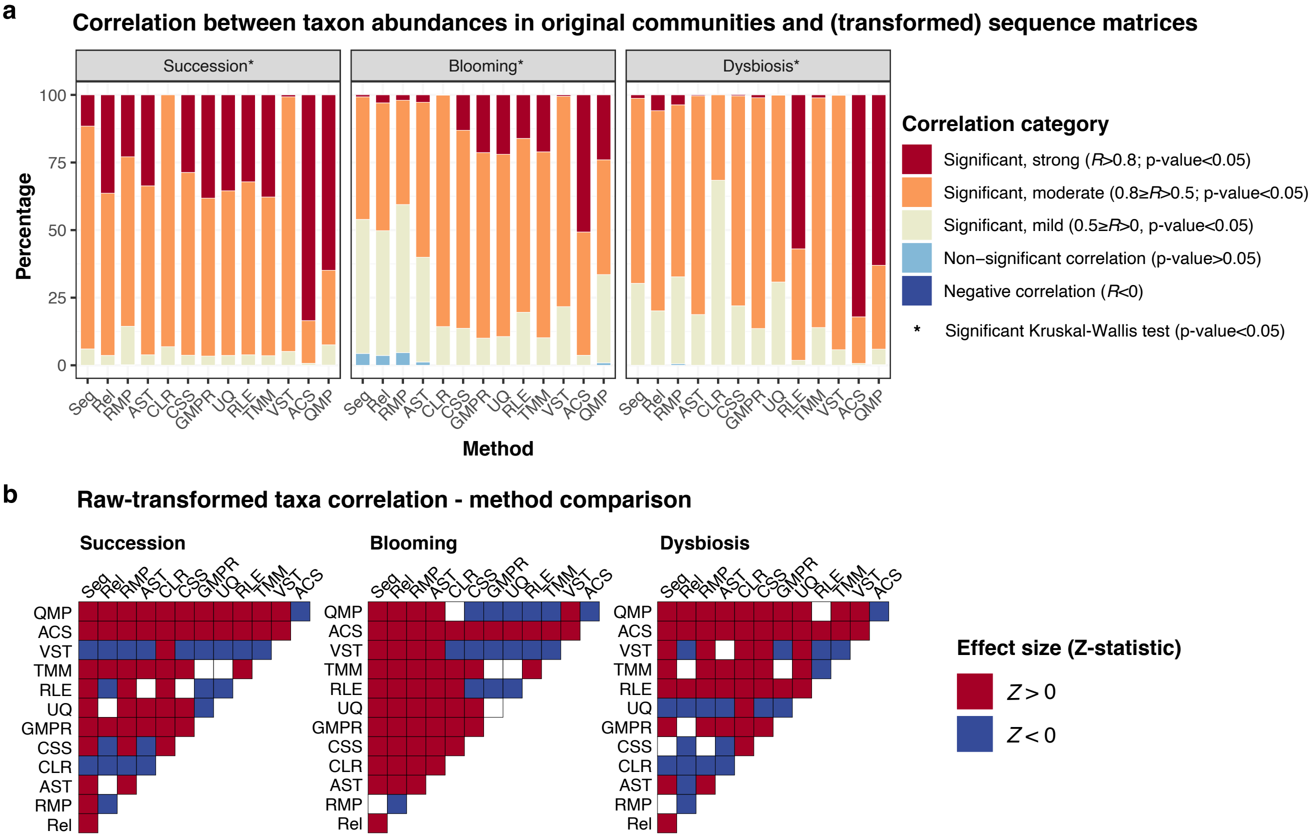

Supplementary Figure 4. Correlation of taxa abundances between synthetic communities and transformed matrices. a. Correlation between taxa abundances in the synthetic communities and (transformed) sequence matrices (Pearson). Correlations are classified in five categories: significant, strong (*R*>0.8, p-value<0.05); significant, moderate (0.8≥*R*>0.5 p-value<0.05); significant, mild (0.5≥*R*>0, p-value<0.05); non-significant (p-value≥0.05); and negative (0>*R*, p-value<0.05). Stacked barplots represent the percentage of correlations falling into each of the categories for each method and scenario (for *n*=10 simulated matrices per scenario). *, Kruskal-Wallis p-value<0.05 (Supplementary Table 5). **b.** Evaluation of method performance based on distribution of correlation coefficients (*R*) determined in panel (a) (pairwise Dunn test). Significant (p-value<0.05) comparisons after multiple testing correction are colored, with colors representing the sign of the effect size (Z-statistic) of each pairwise comparison. A red (blue) color indicates that the method in the corresponding row has a higher (lower) value than the method in the corresponding column. Seq, sequencing data; Rel, relative abundances; RMP, relative microbiome profiling; AST, arcsine square root transformation; CLR, centered log-ratio; CSS, cumulative sum scaling; GMPR, geometric mean of pairwise ratios; UQ, upper quartile; RLE, relative log expression; TMM, trimmed mean of M-values; VST, variance-stabilizing transformation; ACS, absolute count scaling; QMP, quantitative microbiome profiling.

Supplementary Figure 5

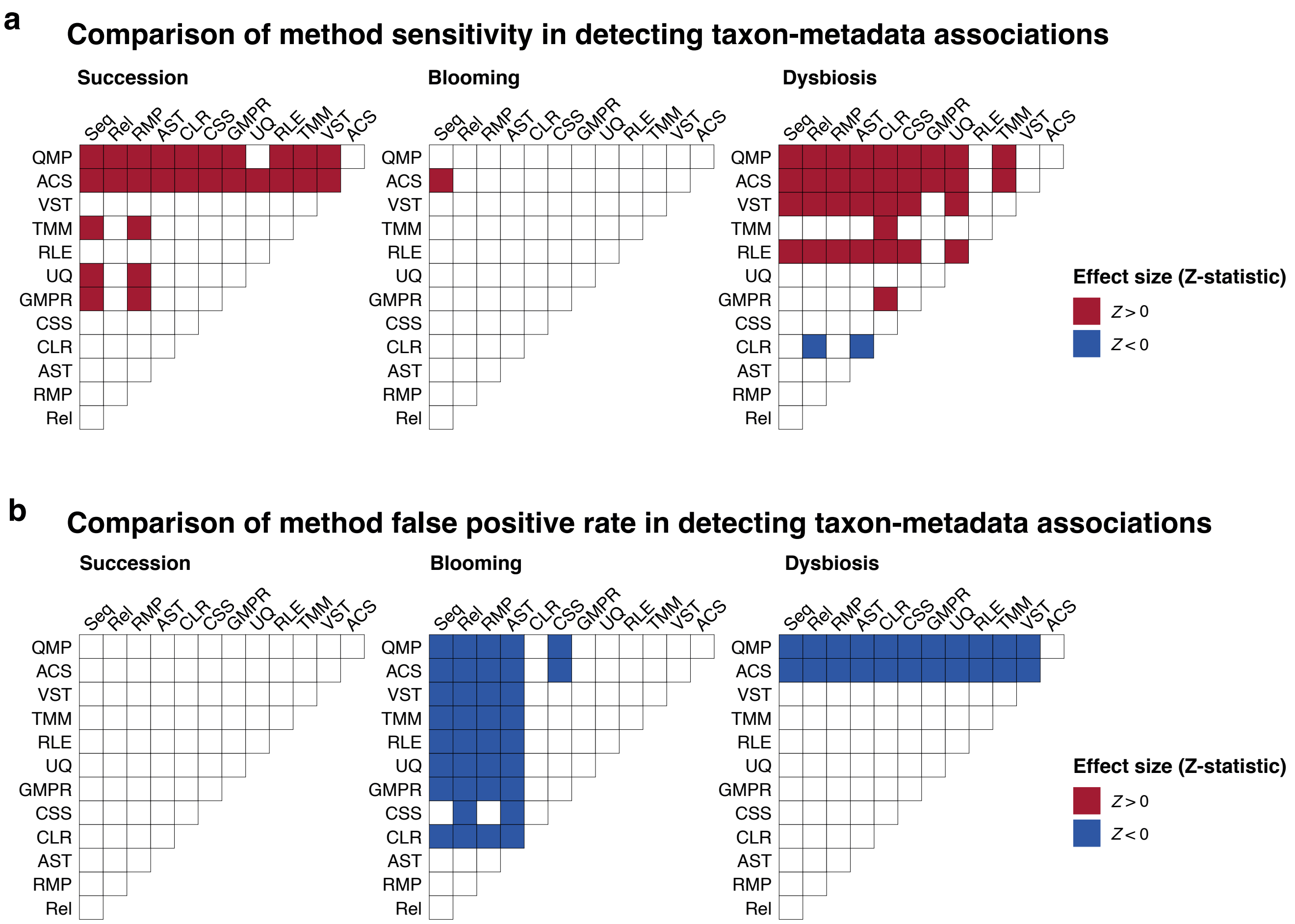

Supplementary Figure 5. Sensitivity and false positive rate of transformation methods in recovering taxon-metadata associations. a. Evaluation of method sensitivity based on Figure 4a classifications (pairwise Dunn test; Supplementary Table 6). Significant (p-value<0.05) comparisons after multiple testing correction are colored, with colors representing the sign of the effect size (Z-statistic) of each pairwise comparison. A red (blue) color indicates that the method in the corresponding row has a higher (lower) value than the method in the corresponding column. **b.** As in (a) for false positive rates. Seq, sequencing data; Rel, relative abundances; RMP, relative microbiome profiling; AST, arcsine square root transformation; CLR, centered log-ratio; CSS, cumulative sum scaling; GMPR, geometric mean of pairwise ratios; UQ, upper quartile; RLE, relative log expression; TMM, trimmed mean of M-values; VST, variance-stabilizing transformation; ACS, absolute count scaling; QMP, quantitative microbiome profiling.

Supplementary Figure 6

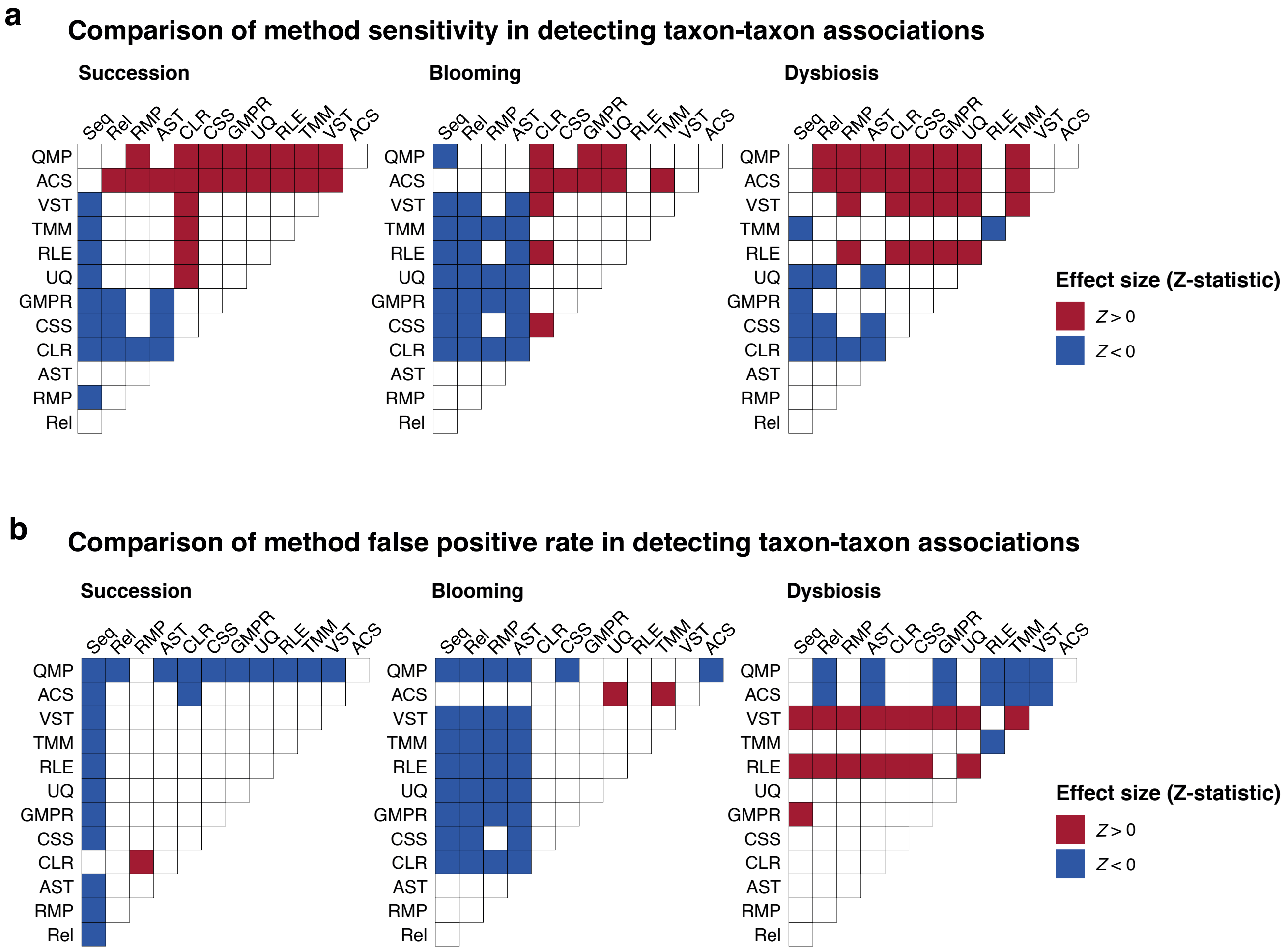

Supplementary Figure 6. Sensitivity and false positive rate of transformation methods in recovering taxon-taxon associations. a. Evaluation of method sensitivity based on Figure 4d classifications (pairwise Dunn test; Supplementary Table 8). Significant (p-value<0.05) comparisons after multiple testing correction are colored, with colors representing the sign of the effect size (Z-statistic) of each pairwise comparison. A red (blue) color indicates that the method in the corresponding row has a higher (lower) value than the method in the corresponding column. **b.** As in (a) for false positive rates. Seq, sequencing data; Rel, relative abundances; RMP, relative microbiome profiling; AST, arcsine square root transformation; CLR, centered log-ratio; CSS, cumulative sum scaling; GMPR, geometric mean of pairwise ratios; UQ, upper quartile; RLE, relative log expression; TMM, trimmed mean of M-values; VST, variance-stabilizing transformation; ACS, absolute count scaling; QMP, quantitative microbiome profiling.

Supplementary figure 7

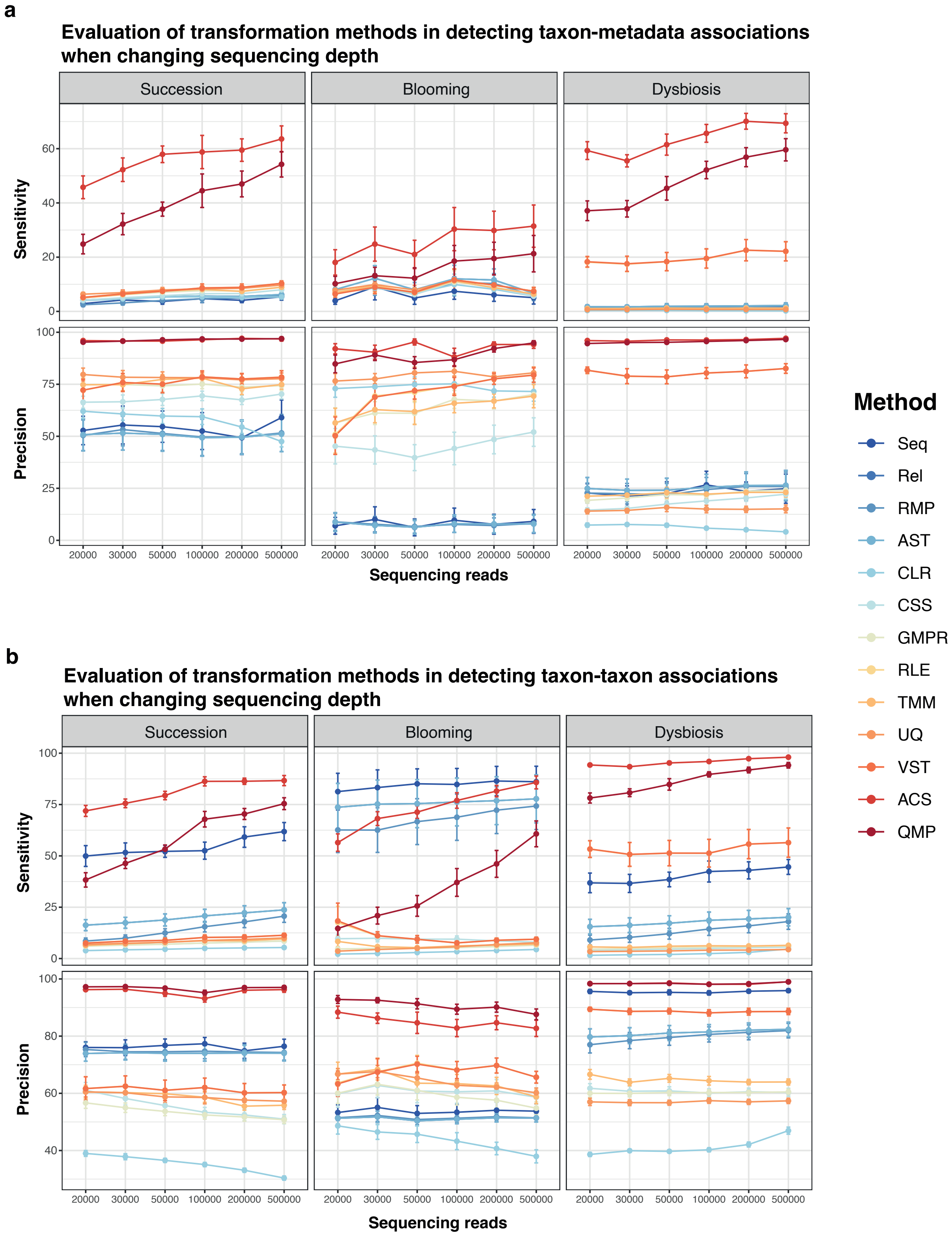

Supplementary Figure 7. Effect of sequencing depth variation on the performance of transformation methods. a. Effect of sequencing depth variation on sensitivity and precision of recovery of taxon-metadata associations by transformation methods. Error bars represent the standard error of the mean for each of the points. **b.** As in (a) for taxon-taxon associations. Seq, sequencing data; Rel, relative abundances; RMP, relative microbiome profiling; AST, arcsine square root transformation; CLR, centered log-ratio; CSS, cumulative sum scaling; GMPR, geometric mean of pairwise ratios; UQ, upper quartile; RLE, relative log expression; TMM, trimmed mean of M-values; VST, variance-stabilizing transformation; ACS, absolute count scaling; QMP, quantitative microbiome profiling. For (a,b), n=10 matrices for each scenario and sequencing depth evaluated.

Supplementary figure 8

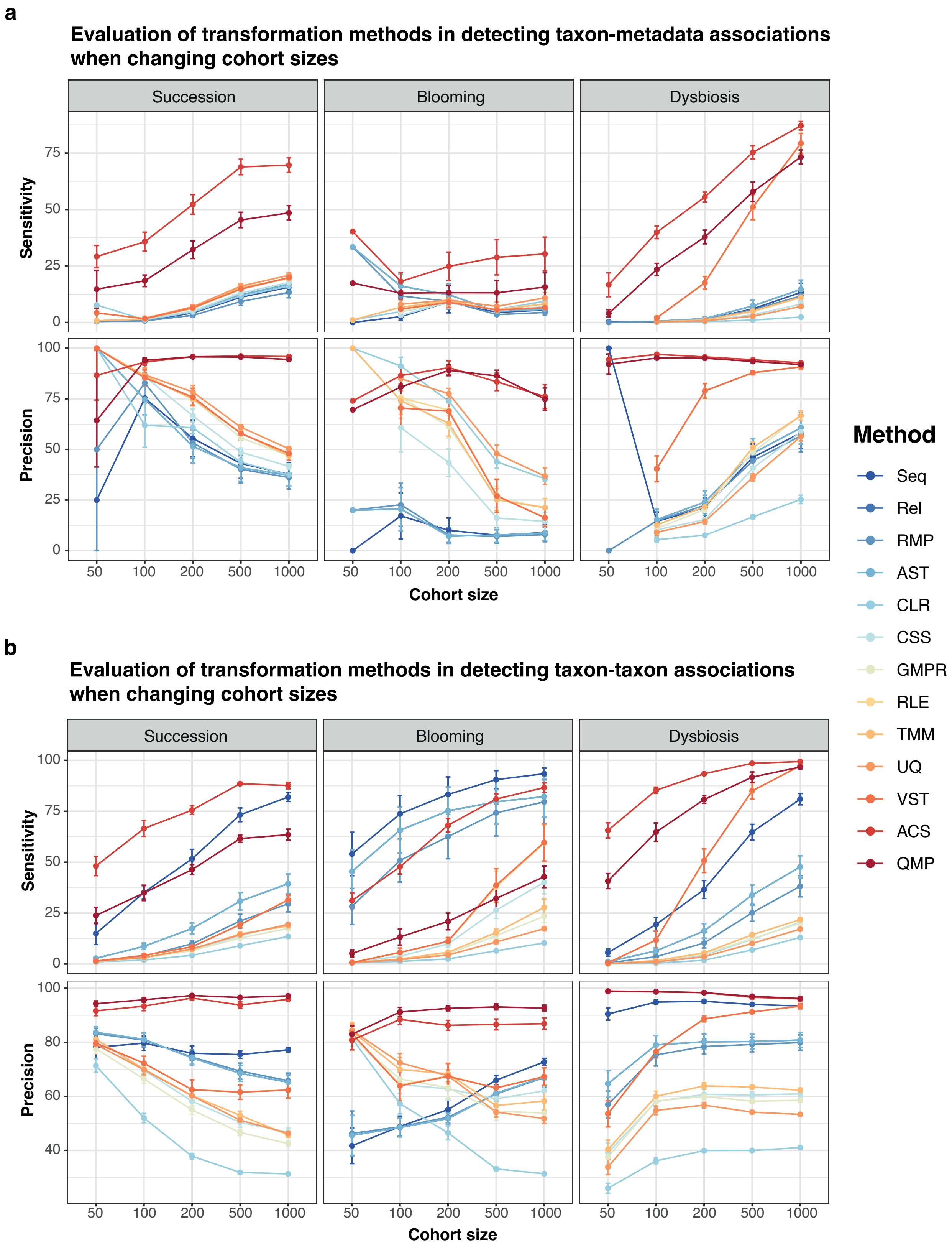

Supplementary Figure 8. Effect of cohort size variation on the performance of transformation methods. a. Effect of cohort size variation on sensitivity and precision of recovery of taxon-metadata associations by transformation methods. Error bars represent the standard error of the mean for each of the points. **b.** As in (a) for taxon-taxon associations. Seq, sequencing data; Rel, relative abundances; RMP, relative microbiome profiling; AST, arcsine square root transformation; CLR, centered log-ratio; CSS, cumulative sum scaling; GMPR, geometric mean of pairwise ratios; UQ, upper quartile; RLE, relative log expression; TMM, trimmed mean of M-values; VST, variance-stabilizing transformation; ACS, absolute count scaling; QMP, quantitative microbiome profiling. For (a,b), *n*=10 matrices for each scenario and sequencing depth evaluated.

Supplementary Figure 9

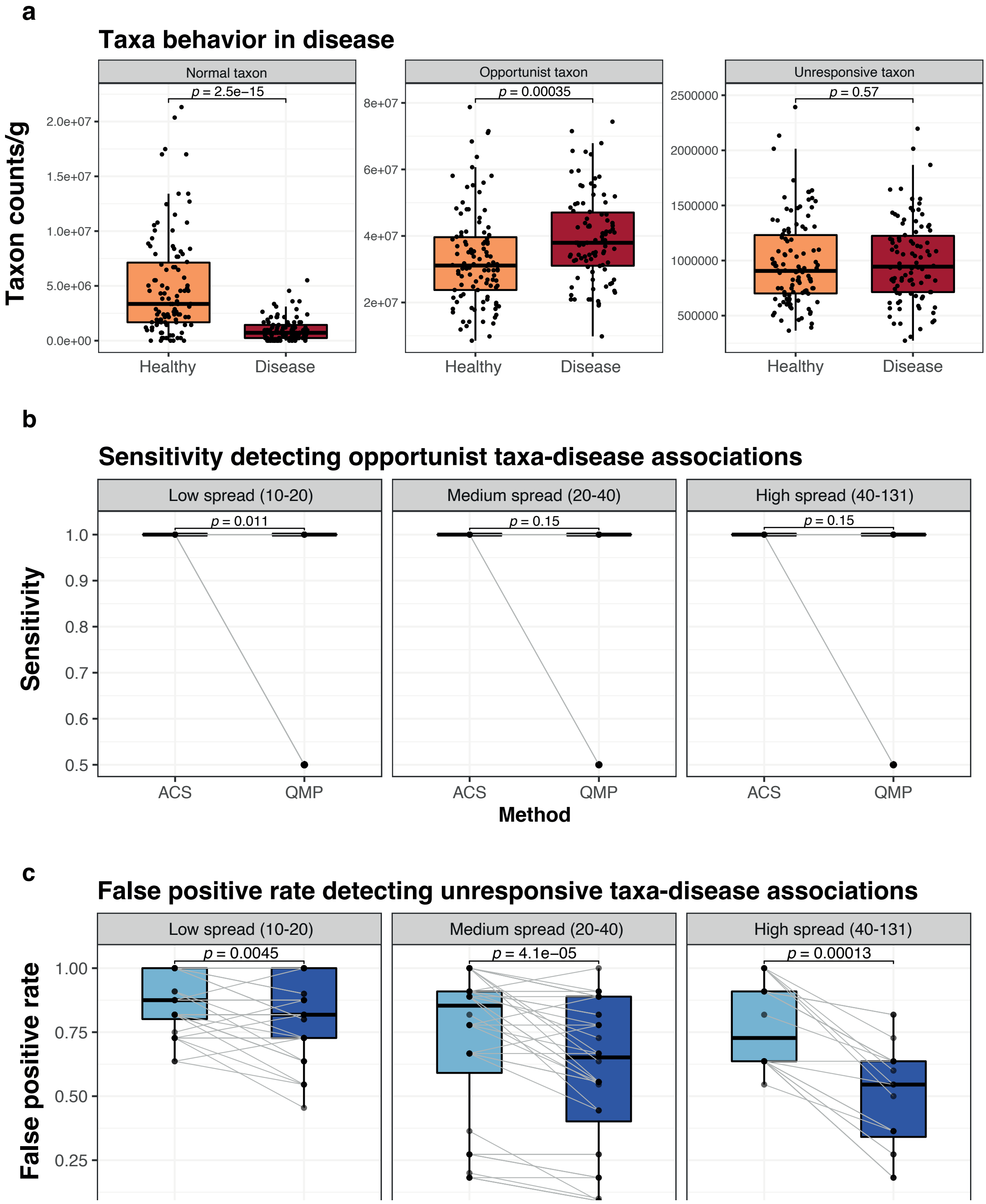

Supplementary Figure 9. Comparative performance of quantitative methods in detecting associations with disease. a. Visualization of different taxa behavior in patients and controls. The majority of taxa are more abundant in the control group (left panel), whilst opportunist taxa are associated to the patient group (mid panel) and unresponsive taxa show no significant association to either (right panel; Wilcoxon rank sum test). Representation of one of the simulated dysbiosis matrices (randomly chosen, same as in Figure 5a; *n*=200 samples, distributed in 93 healthy controls and 107 diseased samples). **b.** Sensitivity

values for the opportunist taxon-disease association in ACS and QMP at different sequencing depths (Wilcoxon signed-rank test). **c.** False positive rate introduced by the quantitative methods at different sequencing depths, specifically focusing on the unresponsive taxa (Wilcoxon signed-rank test). In (b,c), simulated synthetic community matrices were classified in three groups corresponding to the different spreads in microbial loads in the population (low spread, left panel: *n*=40 sequencing matrices simulated from 4 synthetic communities; medium spread, mid panel: *n*=40 sequencing matrices simulated from 4 synthetic communities; high spread, right panel: *n*=20 sequencing matrices simulated from 2 synthetic communities). In (a,b,c), the boxplots extend from the first to the third quartile of the distribution, with the line indicating the median. The whiskers cover from the quartiles to the last data point within 1.5x the interquartile range, with outliers depicted as individual points. ACS, absolute count scaling; QMP, quantitative microbiome profiling.
