# Supplementary material for: Benchmarking microbiome transformations favors experimental quantitative approaches to address compositionality and sampling depth biases
Source: Nat Commun. 2021 Jun 11;12:3562. doi: 10.1038/s41467-021-23821-6 (PMC8196019; doi:10.1038/s41467-021-23821-6)
Supplement: Supplementary file 2 — Description of Additional Supplementary Files [file 41467_2021_23821_MOESM2_ESM.docx]

**Description of Additional Supplementary Files**

**File Name:** Supplementary Data 1

**Description:** Simulated synthetic communities: matrix statistics. Summary of the 30 simulated matrices generated for this study, including the simulated scenario, the opportunist/unresponsive/bloomer taxa if applicable, the microbial loads (real and estimated, i.e. simulated mimicking flow cytometry data), richness and evenness of the communities.

**File Name:** Supplementary Data 2

**Description:** Correlation of alpha diversity indices between synthetic communities and transformed data. Kruskal-Wallis test and, if applicable, post-hoc pairwise Dunn test comparing the correlation values between the simulated synthetic communities and the transformed data for 4 alpha diversity indices (Observed richness, Chao1 richness estimator, Shannon Diversity and Simpson 1-D diversity index).

**File Name:** Supplementary Data 3

**Description:** Correlation of alpha diversity and microbial load for synthetic communities and transformed data. Kruskal-Wallis test and, if applicable, post-hoc pairwise Dunn test comparing the correlation values between the microbial loads in the simulated synthetic communities and 4 alpha diversity indices (Observed richness, Chao1 richness estimator, Shannon Diversity and Simpson 1-D diversity index), both for the simulated synthetic communities ("Real") and the transformed data.

**File Name:** Supplementary Data 4

**Description:** Correlations of taxa abundances between synthetic communities and transformed data. Kruskal-Wallis test and, if applicable, post-hoc pairwise Dunn test comparing the correlation values of individual taxa abundances between the simulated synthetic communities and the transformed data.

**File Name:** Supplementary Data 5

**Description:** Comparison of method performance in detecting taxon-microbial load associations. Kruskal-Wallis test and, if applicable, post-hoc pairwise Dunn test comparing the sensitivity, precision and false positive rate of all the transformations in detecting taxon-microbial load associations.

**File Name:** Supplementary Data 6

**Description:** Comparison of method performance in detecting taxon-metadata associations. Kruskal-Wallis test and, if applicable, post-hoc pairwise Dunn test comparing the sensitivity, precision and false positive rate of all the transformations in detecting taxon-metadata associations.

**File Name:** Supplementary Data 7

**Description:** Statistics on taxon-metadata associations. Raw values of true positives, true negatives, false positives, false negatives and discordant associations detected in taxon-metadata associations; as well as sensitivity, precision and false positive rate for each of the transformation in the different scenarios. Data is calculated for all associations altogether, and when applicable, also for the specific taxa (bloomer/unresponsive/opportunist).

**File Name:** Supplementary Data 8

**Description:** Comparison of method performance in detecting taxon-taxon associations. Kruskal-Wallis test and, if applicable, post-hoc pairwise Dunn test comparing the sensitivity, precision and false positive rate of all the transformations in detecting taxon-taxon associations.

**File Name:** Supplementary Data 9

**Description:** Statistics on taxon-taxon associations. Raw values of true positives, true negatives, false positives, false negatives and discordant associations detected in taxon-taxon associations; as well as sensitivity, precision and false positive rate for each of the transformation in the different scenarios. Data is calculated for all associations altogether, and when applicable, also for the specific taxa (bloomer/unresponsive/opportunist).

**File Name:** Supplementary Data 10

**Description:** Parameter exploration results: method performance when changing sequencing depths. Sensitivity, precision and false positive rate values for all transformations evaluated, simulating different sequencing depths.

**File Name:** Supplementary Data 11

**Description:** Parameter exploration results: method performance when changing cohort size. Sensitivity, precision and false positive rate values for all transformations evaluated, simulating different cohort sizes.

**File Name:** Supplementary Data 12

**Description:** Comparison between QMP and ACS across sequencing depths and spread in microbial loads. Sensitivity, precision and false positive rate values for both experimental methods, QMP and ACS, in detecting taxon-disease associations in the dysbiosis scenario, at various sequencing depths.
