# Supplementary material for: Benchmarking microbiome transformations favors experimental quantitative approaches to address compositionality and sampling depth biases
Source: Nat Commun. 2021 Jun 11;12:3562. doi: 10.1038/s41467-021-23821-6 (PMC8196019; doi:10.1038/s41467-021-23821-6)
Supplement: Supplementary file 15 — Reporting Summary [file 41467_2021_23821_MOESM15_ESM.pdf]

nature research

Corresponding author(s): Jeroen Raes

Last updated by author(s): May 7, 2021

Reporting Summary

Nature Research wishes to improve the reproducibility of the work that we publish. This form provides structure for consistency and transparency in reporting. For further information on Nature Research policies, see our Editorial Policies and the Editorial Policy Checklist.

Statistics

For all statistical analyses, confirm that the following items are present in the figure legend, table legend, main text, or Methods section.

n/a	Confirmed	
☐	☒	The exact sample size (n) for each experimental group/condition, given as a discrete number and unit of measurement
☐	☒	A statement on whether measurements were taken from distinct samples or whether the same sample was measured repeatedly
☐	☒	The statistical test(s) used AND whether they are one- or two-sided<br>*Only common tests should be described solely by name; describe more complex techniques in the Methods section.*
☐	☒	A description of all covariates tested
☐	☒	A description of any assumptions or corrections, such as tests of normality and adjustment for multiple comparisons
☐	☒	A full description of the statistical parameters including central tendency (e.g. means) or other basic estimates (e.g. regression coefficient) AND variation (e.g. standard deviation) or associated estimates of uncertainty (e.g. confidence intervals)
☐	☒	For null hypothesis testing, the test statistic (e.g. F, t, r) with confidence intervals, effect sizes, degrees of freedom and P value noted<br>*Give P values as exact values whenever suitable.*
☒	☐	For Bayesian analysis, information on the choice of priors and Markov chain Monte Carlo settings
☒	☐	For hierarchical and complex designs, identification of the appropriate level for tests and full reporting of outcomes
☐	☒	Estimates of effect sizes (e.g. Cohen's d, Pearson's r), indicating how they were calculated

Our web collection on statistics for biologists contains articles on many of the points above.

Software and code

Policy information about availability of computer code

Data collection	Simulated data was generated as described in methods section using Matlab R2015b and R (v3.6.3)
Data analysis	All data analyses were performed in R (v3.6.3). For data preprocessing, analyses and visualization, the following R packages were used: tidyverse (v1.3.0), gdata (v2.18.0), GMPR (v0.1.3), CoDaSeq (v0.99.6), phyloseq (v1.34.0), DESeq2 (v1.30.1), metagenomeSeq (v1.32.0), edgeR (v3.32.1), zCompositions (v1.3.4), rstatix (v0.7.0), ggpubr (v0.4.0), ggsignif (v0.6.1), inlmisc (v0.5.2) and RColorBrewer (v1.1.2).<br><br>All analysis scripts are made available in https://raeslab.org/software/BMT/index.html and also deposited in Zenodo with the following unique identifier: DOI:10.5281/zenodo.4719508

For manuscripts utilizing custom algorithms or software that are central to the research but not yet described in published literature, software must be made available to editors and reviewers. We strongly encourage code deposition in a community repository (e.g. GitHub). See the Nature Research guidelines for submitting code & software for further information.

Data

Policy information about availability of data

All manuscripts must include a data availability statement. This statement should provide the following information, where applicable:

- Accession codes, unique identifiers, or web links for publicly available datasets
- A list of figures that have associated raw data
- A description of any restrictions on data availability

Simulated matrices presented in this manuscript are available in https://raeslab.org/software/BMT/index.html and also deposited in Zenodo with the following unique identifier: DOI:10.5281/zenodo.4719508

Field-specific reporting

Please select the one below that is the best fit for your research. If you are not sure, read the appropriate sections before making your selection.

- [x] Life sciences
- [ ] Behavioural & social sciences
- [ ] Ecological, evolutionary & environmental sciences

For a reference copy of the document with all sections, see nature.com/documents/nr-reporting-summary-flat.pdf

Life sciences study design

All studies must disclose on these points even when the disclosure is negative.

Sample size	For the majority of analyses presented in the manuscript, a sample size of N=200 samples for each simulated matrix was used. This sample size is not predetermined, but as part of this study, the effect of sample size in the different transformations was evaluated (with sample sizes ranging from N=50 to N=1000), providing ground for sample size estimations for future microbiome studies.<br>Additionally, for each scenario, 10 different matrices were evaluated. This sample size was also not predetermined but has proven to be sufficient to discriminate the differences in sensitivity, precision and false positive rate across the methods evaluated.
Data exclusions	Except when indicated otherwise, taxa with a prevalence lower than 50% in the sequencing matrices were discarded of all the analyses to reduce data sparseness. This exclusion was not predetermined and established ad-hoc for the analyses presented in this manuscript to minimize table sparseness.
Replication	There are no experimental findings in this study as it is purely computational and based in simulated data. Therefore, reproducibility and replication are ensured by making the code used to produce these results available.
Randomization	This study refers to simulated data only, and therefore samples in the simulated matrices were not allocated to any experimental group. However, random metadata variables were generated for each simulated matrix, with or without a pre-specified correlation with specific taxa or with total microbial loads. Details of the generation of such metadata matrices can be found in the methods section and in the publicly available code to reproduce the findings of this study.
Blinding	The findings in this study refer only to simulated data, therefore blinding of group allocation of samples to the investigators is not applicable.

Reporting for specific materials, systems and methods

We require information from authors about some types of materials, experimental systems and methods used in many studies. Here, indicate whether each material, system or method listed is relevant to your study. If you are not sure if a list item applies to your research, read the appropriate section before selecting a response.

Materials & experimental systems

n/a	Involved in the study	
x		Antibodies
x		Eukaryotic cell lines
x		Palaeontology and archaeology
x		Animals and other organisms
x		Human research participants
x		Clinical data
x		Dual use research of concern

Methods

n/a	Involved in the study	
x		ChIP-seq
x		Flow cytometry
x		MRI-based neuroimaging
